# Taxonomic review of *Cratocerus* Dejean, 1829 (Coleoptera, Carabidae) with the description of six new species

**DOI:** 10.3897/zookeys.416.6455

**Published:** 2014-06-17

**Authors:** Traci L. Grzymala, Kipling W. Will

**Affiliations:** 1 ESPM Department and Essig Museum of Entomology, University of California, Berkeley, CA 94720

**Keywords:** Pterostichinae, Harpalinae, Cratocerini, Cratocerina, revision, key

## Abstract

A diagnosis of the South and Central American genus *Cratocerus* Dejean (Coleoptera: Carabidae) and a key to all species is provided. Eight species are recognized including six species that are newly described: *Cratocerus sinesetosus* **sp. n.** from French Guiana and Peru; *Cratocerus multisetosus* **sp. n.** from Costa Rica and Panama; *Cratocerus tanyae* **sp. n.** from Costa Rica, Guatemala, and Mexico; *Cratocerus indupalmensis* **sp. n.** a species widely distributed throughout Central and South America; *Cratocerus kavanaughi* **sp. n.** from French Guiana and Peru; and *Cratocerus culpepperi* **sp. n.** from Peru. A lectotype for *Cratocerus sulcatus* Chaudoir is designated. Habitus images are provided along with illustrations and images of male genitalia, female genitalia, and diagnostic morphological characters.

## Introduction

The genus *Cratocerus* (Coleoptera: Carabidae) was originally described by Dejean (1829) based on two specimens collected from Brazil. Dejean described these as *Cratocerus monilicornis* and placed the genus within his first division of the Carabidae characterized by moniliform antennae. This first division included two additional genera, *Somoplatus* Dejean and *Daptus* Fischer von Waldheim, *Cratocerus* was separated from these based on the oval shape of the second labial palpomere. Chaudoir (1852) described an additional species of the genus, *Cratocerus sulcatus* from specimens collected in Mexico. Lacordaire (1854) placed *Cratocerus* in his group Cratocérides, which included the genera of Dejean's, first division along with *Cyclosomus* Latreille, *Pachytrachelus* Chaudoir, *Microderes* Faldermann, *Geopinus* LeConte, *Batoscelis* Dejean, *Agonoderus* Dejean, *Brachidius* Chaudoir, *Macracanthus* Chaudoir, *Euryderus* LeConte (as *Nothopus* LeConte), *Amblygnathus* Dejean, and *Polpolchila* Solier (as *Melanotus* Dejean). Murray (1858) placed this grouping at the rank of family, Cratoceridae, and included the newly erected African genus *Diatypus* Murray (currently considered a subgenus within *Notiobia* Perty). Chaudoir (1872) later dismantled this grouping and kept only *Cratocerus*, *Brachidius*, and *Catapiesis* Solier (as *Basoleia* Westwood) within the Cratocérides. Though this restricted Cratocérides was ignored by Chapuis (1876), Bertkau (1873) and Horn (1881) agreed with Chaudoir's, restructuring and recognized the same grouping as Tribe XXXVIII, the Cratocerini.

Bates (1882) separated out *Catapiesis* along with *Homalomorpha* Brulle as their own subfamily, the Catapiesinae, based on the truncate elytra of the species, which left *Cratocerus* and *Brachidius* to be grouped together as their own subfamily, the Cratocerinae. Sloane (1923) did not adopt the classification proposed by Bates, but he was not able to examine specimens of *Cratocerus* during his studies and instead based the tribal characters on *Catapiesis* exemplars. Csiki (1929) and Blackwelder (1944) kept the constitution of Bates’ subfamilies the same, but regarded these as subtribes within Pterostichini – Catapiesi and Cratoceri. Reichardt (1973) removed the Catapiesi from the Pterostichini, placing *Catapiesis* and *Homalomorpha* as their own tribe, the Catapiesini. *Cratocerus* therefore became a monogeneric subtribe, the Cratocerina, within the Pterostichini (Whitehead and Ball 1974, Reichardt 1977, Straneo 1979). In the checklist of Lorenz (2005), the tribe Cratocerini is considered to be within the subfamily Pterostichinae and is composed of the two subtribes Catapiesina (*Catapiesis* and *Homalomorpha*) and Cratocerina s. str. (*Cratocerus*, *Brachidius*, and *Oxyglychus* Straneo). Bouchard et al. (2011) alternatively placed the tribes Cratocerini and Catapiesini within the subfamily Harpalinae.

Very little is known regarding the natural history of either the larval or adult stages of any *Cratocerus* species. Emden (1942) described a larva of *Cratocerus*, which was found in a rotting stump of a banana tree in Costa Rica, and allied this larva with those he had seen of the African genus *Caelostomus* MacLeay. Label data of adult specimens indicate that they are often collected underneath decaying tree bark.

## Materials and methods

A Leica MZ12.5 stereomicroscope was used to examine 185 adult specimens during this study. Length and ratio measurements were obtained using a calibrated ocular micrometer. When available, ten specimens were measured with at least one from each locality for each measurement to encompass the range of variability. When less than ten were available, measurements for all specimens were taken. Measurements listed include apparent body length (ABL), which was measured from the apex of the labrum to the apex of the elytra; total width (TW), which was measured across the widest point of the elytra; head width (HW) measured across the supraorbital ridges; head length (HL) measured from apex of labrum to base of the head; pronotal width (PW) taken across the widest point of the pronotum, usually anterad to center; pronotal length (PL), measured at pronotal midline from apex to base; and elytral length (EL) measured from humeri to elytral apex.

Habitus images were taken with a BK Lab Imaging system (Visionary Digital) and montaged in Helicon Focus 5.3. At least one male and one female specimen were dissected from each collection locality. Male dissections were performed by first using a hooked insect pin to extract the terminalia, which were then placed into a warm 10% KOH solution for 15–20 minutes to clear surrounding tissue. Dissections were then neutralized in 10% acetic acid and washed in water. For female specimens, the entire abdomen was removed, softened, and subsequently cleared in warm 10% KOH for 20–25 minutes. The genitalia were then removed and placed in chlorazol black for 5–10 minutes in order to stain membranous structures. Line drawings were made using a camera lucida and then digitally inked in Adobe Illustrator. Scanning electron micrographs were obtained at the California Academy of Sciences using a Leo 1450VP. Specimens were air dried and coated with gold/palladium using a Denton Vacuum Desk-II prior to imaging. Maps were created using the Cartographer package version 1.31 in Mesquite version 2.75 (build 566) (D. R. Maddison and Maddison 2011; W. P. Maddison and Maddison 2011) using portions of the World, shaded relief and colored height base image from NASA (NASA/JPL/NGA 2004), which was modified for color contrast and cropped to fit.

Verbatim label data for primary type material are listed for each species. A slash (/) indicates separation of lines within one label and a double slash (//) indicates different labels for the specimen. All specimens evaluated in this study were determined to species and bear an identification label. Specimens were borrowed from and will be deposited in the following institutions: American Museum of Natural History, New York, NY (AMNH); Natural History Museum, London, UK (BMNH); Carnegie Museum of Natural History, Pittsburgh, PA (CMNH); Canadian National Collections of Insects, Ottawa, Canada (CNCI); Cornell University Insect Collection, Cornell, NY (CUIC); Museum of Comparative Zoology, Essig Museum of Entomology, Berkeley, CA (EMEC); Harvard University, Cambridge, MA (MCZC); Museo Civico di Storia Naturale, Milan, Italy (MSNM); Muséum National d’Histoire Naturelle, Paris, France (MNHN); and the National Museum of Natural History, Washington, DC (NMNH). All specimen data have been entered into the Essig Museum of Entomology Collection (EMEC) database and records can be accessed online using the EMEC numbers listed for each specimen (http://essigdb.berkeley.edu/query_specimens.html).

The Phylogenetic Species concept *sensu* Wheeler and Platnick (2000) is used in this study where species are the smallest aggregation of specimens diagnosable by a unique set of character states.

## Taxon treatments

### Cratocerus

Taxon classificationAnimaliaColeopteraCarabidae

Dejean 1829

Cratocerus Dejean 1829: 3, 12–13; Hope 1838: 84; Chaudoir 1852: 77-79; Lacordaire 1854: 257, 263-264; Gemminger and Harold 1868: 250; Chaudoir 1872: 17–18; Bertkau 1873: 269; Chapuis 1876: 269; Horn 1881: 163; Bates 1882: 90; Sloane 1923: 249; Csiki 1929: 493; Blackwelder 1944: 34; Reichardt 1973: 324–325; Whitehead and Ball 1974: 595; Reichardt 1977: 407; Straneo 1977: 108, 115; Straneo 1979: 346, 352; Lorenz 2005: 248; Bouchard et al. 2011: 124.

#### Type species:

*Cratocerus monilicornis* Dejean 1829: 14–15.

#### Diagnosis.

Medium to small, rufopiceous to black beetles with a compact, convex body form. Head is relatively broad with prominent eyes and moniliform antennae. Elytral striae are distinctly sulcate. Tergite VIII is divided into hemitergites and those divided into epitergites. This combination of characters distinguishes members of *Cratocerus* from all other carabids within their range including similar genera, e.g. *Catapiesis* and *Moriosomus* Motschulsky.

#### Description.

*Microsculpture*. Head dull in appearance with isodiametric microsculpture; pronotum shining with markedly transverse microsculpture; procoxa distinctly granulate isodiametric; abdominal sternites with transverse microsculpture. *Head*. Prominent supraorbital ridge, occasionally with slight rugosity; one or two pairs of supraorbital setae. Frons glabrous. Fronto-clypeal suture distinct. Clypeus with pair of setae present mediolaterally. Labrum with six anterior marginal setae, outer pair length 1/3 × middle pair, middle pair length 1/3 × inner pair; apex of labrum often slightly medially emarginate. Mentum with one pair of setae and prominent medial tooth, submentum with two pairs of setae; transverse suture between mentum and submentum complete. Scape glabrous except for one dorsal seta; pedicel length 1/3 × length of scape, constricted at base with slight apical expansion, with one ring of testaceous setae surrounding apex, antennomere 3 length 1 ½ × that of scape, longer than wide, with several rings of testaceous setae confined to apical 2/3; antennomeres 4–10 transverse and subequal to one another, covered with dense pubescence similarly colored to integument laterally, resulting in matte appearance, midline of antennomeres moderately pubescent; antennomere 11 slightly longer than 10 and broadly rounded apically; antennal length extended to pronotal base. *Thorax*. Pronotal disc 1.1–1.4 × wider than long. Median impression distinct, single basal fovea laterad or pair of fovea laterad with additional fovea overlaying medial impression. Pronotal hind angles sharply denticulate. Elytral length 2.3–2.9 × pronotal length and 1.3–1.6 × elytral width; striae moderately impressed, impunctate or crenulostriate, striae 1, 2, and 7 reaching elytral apex; striae 3 and 4 anastomozing anterad of elytral apex; striae 5 and 6 anastomozing anterad of elytral apex; intervals slightly convex or subtectiform; elytral humeri sharply denticulate; apex rounded, not truncate; plica absent. *Legs*. Protarsomeres 1–4 of males with paired rows of ventral setal pads formed from squamate setae; females with ventral mediolateral spines. Metatrochanter with one basal seta. Metafemur anterior face with one seta basally located and one medially located. *Abdomen*. Abdominal sternites IV–VII glabrous except for a single pair of ambulatory setae; males without setiferous fovea; males and females with single pair of setae on sternite VII. Males with parameres glabrous, left paramere larger than right paramere, left paramere with simple to complex curvature (Fig. 11a–g). Females without medial sclerotized ramus at base of gonocoxa; gonocoxa IX clearly separated into basal and apical aspects, gonocoxite 1 and gonocoxite 2; gonocoxite 2 apex pointed and curved laterad, with short, single, broad lateral ensiform seta and short, single, broad dorsal ensiform seta, apical sensory furrow with two nematiform setae; spermathecal gland lacking.

#### Distribution.

The genus is distributed (Fig. 13) from southern Mexico throughout Central America, and into the South American countries of Colombia, Ecuador, Peru, French Guiana, Brazil, and Argentina.

#### Ecology.

Adults are often collected under the bark of fallen tree branches.

#### Additional literature.

Dejean and Boisduval 1834: 15–17; Schaum 1853: 190; Schaum 1860: 174; Scudder 1882: 81; Emden 1942: 34, 65; Darlington 1962: 507; Freitag 1969: 90; Erwin and Erwin 1976: 219; Erwin 1979: 558; Allen and Ball 1979: 491; Ball and Erwin 1982; 485, 498; Erwin and Sims 1984: 361, 376; Erwin 1990: 40, 54, fig. 53; Erwin 1991: 7; Will et al. 2000: 467; Will 2004: 640.

### Cratocerus monilicornis

Taxon classificationAnimaliaColeopteraCarabidae

Dejean, 1829

Figs 1 , 9a , 11c

Cratocerus monilicornis Dejean 1829: 14-15; Dejean and Boisduval 1834: 17; Hope 1838: 84; Lacordaire 1854: 263-264; Gemminger and Harold 1868: 250; Chaudoir 1872: 18; Csiki 1929: 493; Blackwelder 1944: 34; Straneo 1977: 115; Lorenz 2005: 248.

#### Material.

1 male, labeled: “Museum Paris Montagnes des Orgues Prov de Rio-Janeiro Env. De la Tijuca Massif du Bico Do Papagaio E.R. Wagner 1902 700 m alt. Dicembre” (EMEC654896) borrowed from MNHN; 1 male, labeled: “Brazil, [Santa Catarina] Nova Teutonia, 27°11'S, 52°23'W, [-27.183 -52.383], Fritz Plaumann, 4.xi.1939” (no EMEC# assigned for this specimen, examined at MNSM); 1 female, labeled: “Corcovado Brazil GB 27-IV-1962 J. & B. Bechyne leg.” (EMEC654898) borrowed from CUIC; 1 female, labeled: “Argt. Misiones Bemberg 29-1-45 Hayward-Wilink 60 bach” (EMEC654897) borrowed from MNHN.

#### Diagnosis.

This species is easily distinguished from all other congeners by its relatively large overall body size (9.5–12.0 mm), two pairs of supraorbital setae, and rounded lateral lobes of the mentum.

#### Description.

Size large for the genus, ABL 9.5–12.0 mm, TW 4.0–5.0 mm. *Color*. Head, clypeus, mandibles, pronotum, elytra, and venter rufo-piceous; labrum, antennae, remaining mouthparts, and legs fusco-ferrugineous. *Head*. HL 1.6–1.9 mm, HW 1.4–1.7 mm. Two supraorbital setae present over each eye. Apices of mandibles slightly curved, apices at less than 45 dedgree angle to mandibular midline. Apical maxillary palpomere length 5 × width at base. Mentum with rounded lateral lobes. *Thorax*. Pronotum (Fig. 9a) with distinct median impression, one distinct basal fovea laterad; one lateral seta located on apicolateral pronotal bead; one posterior seta present on posterior bead; pronotal anterior margin convex, greatest width at center; pronotal width 1.3–1.4 × pronotal length. Elytral length 1.4–1.5 × combined elytral width. *Legs*. Protibia with five distinct, stout spines confined to distal half on lateral margin. *Genitalia, male*. Median lobe curved sharply, between forty-five and ninety degrees, with respect to basal 1/3, remaining 2/3 with continuing curvature; not expanded apically; length average for genus. Left paramere complexly curved (Figure 11c). *Genitalia, female*. Spermatheca not examined for this species.

**Figure 1.**
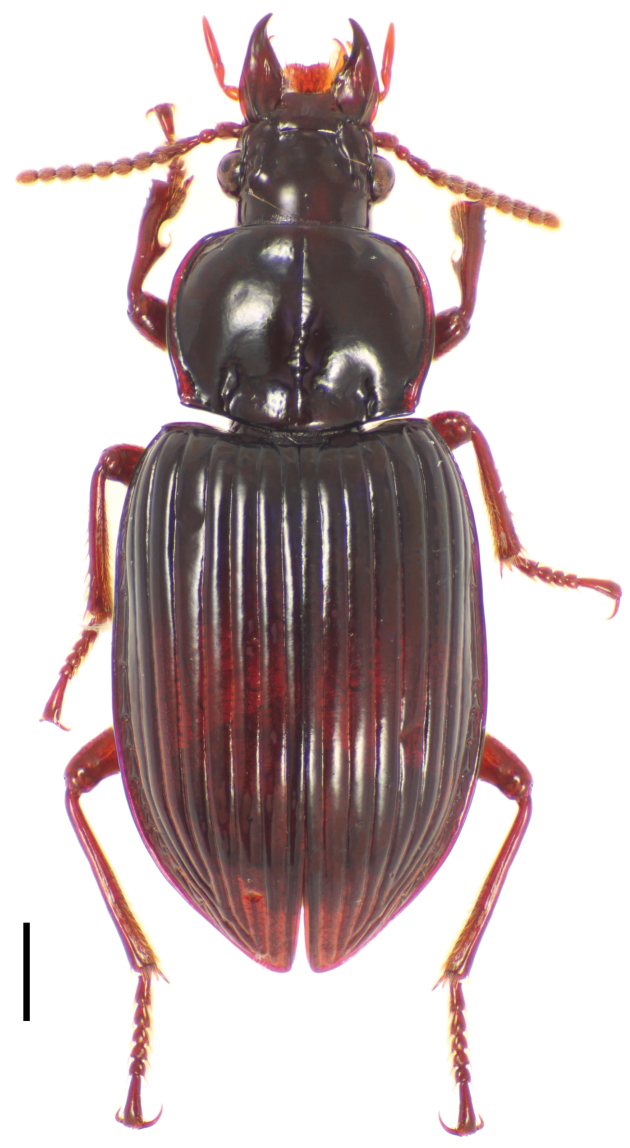
Dorsal habitus image of *Cratocerus monilicornis* Dejean. Scale bar = 1.0 mm.

#### Distribution.

This species is geographically distributed in Brazil and northeastern Argentina (Fig. 13). According to Csiki 1929 and Blackwelder 1944, *Cratocerus monilicornis* has also been recorded from Venezuela, but specimens from this locality were not examined during this study.

#### Additional literature.

Dejean and Boisduval 1832: fig. 3; Ragusa 1887: 210.

### Cratocerus sulcatus

Taxon classificationAnimaliaColeopteraCarabidae

Chaudoir, 1852

Figs 2 , 9b

Cratocerus sulcatus Chaudoir 1852: 77-78; Schaum 1853: 190-191; Gemminger and Harold 1868: 250; Chaudoir 1872: 18; Bates 1882: 91, tab. IV, fig. 18; Csiki 1929: 493; Blackwelder 1944: 34; Reichardt 1977: 407; Lorenz 2005: 248.

#### Type material.

LECTOTYPE [here designated, male, deposited in MNHN] labeled: “Mexique // Sulcatulus / Chaud // Ex. Musaeo / Miniszech // 6yr coll. Minizech // CHD coll. / #3 // UC Berkeley / EMEC / 654900”.

#### Other material.

1 male, labeled: “PERU: San Martin:Hera, 15 km S.E. Moyabamba 860m, VII-1947 F. Woytkowski Coll. Donor Wm. Procter” (EMEC207958) borrowed from AMNH; 1 male, labeled: “Ega Bates coll” (EMEC207859) borrowed from MNHN.

#### Diagnosis.

This species is most easily confused with *Cratocerus sinesetosus* and *Cratocerus multisetosus*, but is distinguished by the one pair of pronotal apicolateral setae whereas *Cratocerus sinesetosus* has no setae at this location and *Cratocerus multisetosus* has two or three pairs of setae at this location.

#### Description.

Size average for the genus, ABL 5.9–6.1 mm, TW 2.6–2.7 mm. *Color*. Head and prothorax brunneopiceous, elytra and abdominal sternites brunneous, legs, antennae, and mouthparts rufotestaceous. *Head*. HL 0.8 mm, HW 0.8–0.9 mm. One supraorbital seta present over each eye. Apices of mandibles slightly curved, apices at less than 45 degree angle to mandibular midline. Apical maxillary palpomere length 5 × width at base. Mentum with triangular lateral lobes. *Thorax*. Pronotum (Fig. 9b) with distinct median impression, one distinct basal fovea laterad; one lateral setae located on apicolateral pronotal bead; one posterior seta present on posterior bead near hind angle; pronotal anterior margin convex; pronotal width 1.3x pronotal length. Elytral length 1.4–1.5x combined elytral width. Elytral intervals prominent, subtectiform. *Legs*. Protibia with three distinct, stout spines confined to distal half of lateral margin; distance between spines 1 and 2 slightly less than distance between spines 2 and 3. *Genitalia, male*. Median lobe curved sharply, between forty-five and ninety degrees, with respect to basal 1/3, remaining 2/3 with continuing curvature; not expanded apically; length average for genus. Left paramere complexly curved into an “S” shape with basal projection, indistinguishable from *Cratocerus multisetosus* (Fig. 11b). *Genitalia, female*. Not examined for this species.

**Figure 2.**
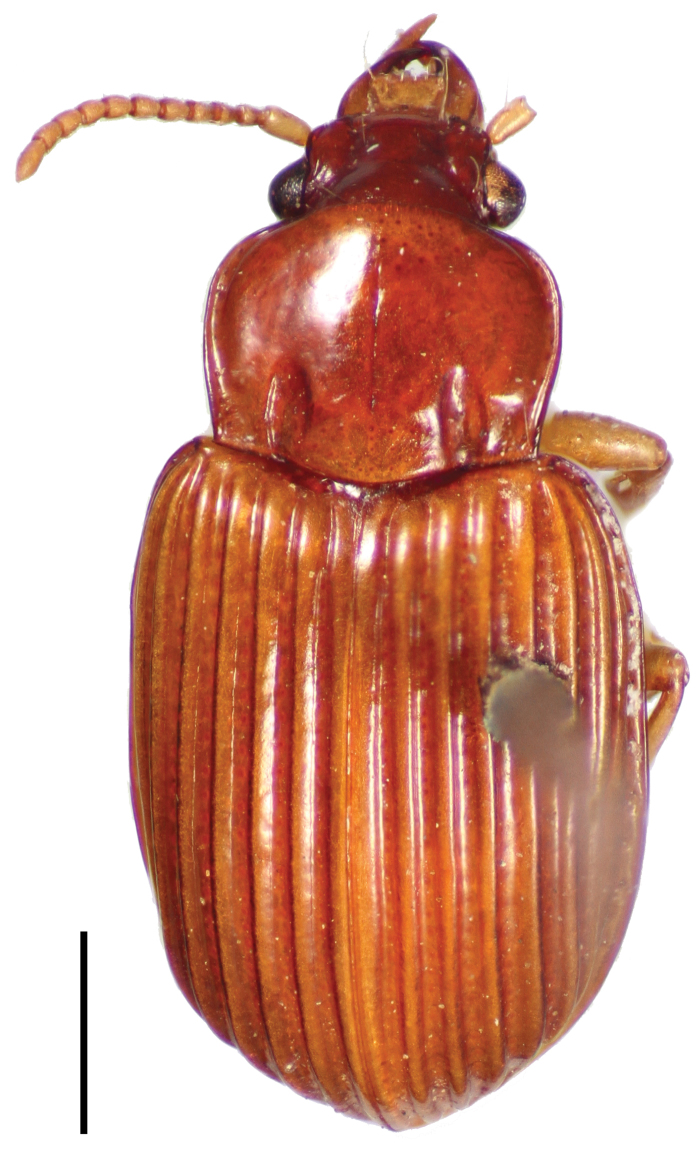
Dorsal habitus image of *Cratocerus sulcatus* Chaudoir, lectotype. Scale bar = 1.0 mm.

#### Distribution.

Figure 13. The lectotype specimen (here designated) for this species is from Mexico, with the type locality having no further restriction at this time. Only two additional specimens were identified as this species -- one from Peru and one from Brazil. These deviate from the type in having slightly less prominent elytral intervals and in the Brazilian specimen, which was smaller, a broader ninth interval near the bases. However, no decisive differences were found and male genitalia were identical. Without additional specimens to examine from across the range, we have tentatively included all three specimens in *Cratocerus sulcatus*.

#### Additional literature.

Emden 1942: 65.

### Cratocerus sinesetosus

Taxon classificationAnimaliaColeopteraCarabidae

Grzymala & Will sp. n.

http://zoobank.org/89EB4B99-B0B5-4B1D-8E23-52AC96847732

**Figure 3.**
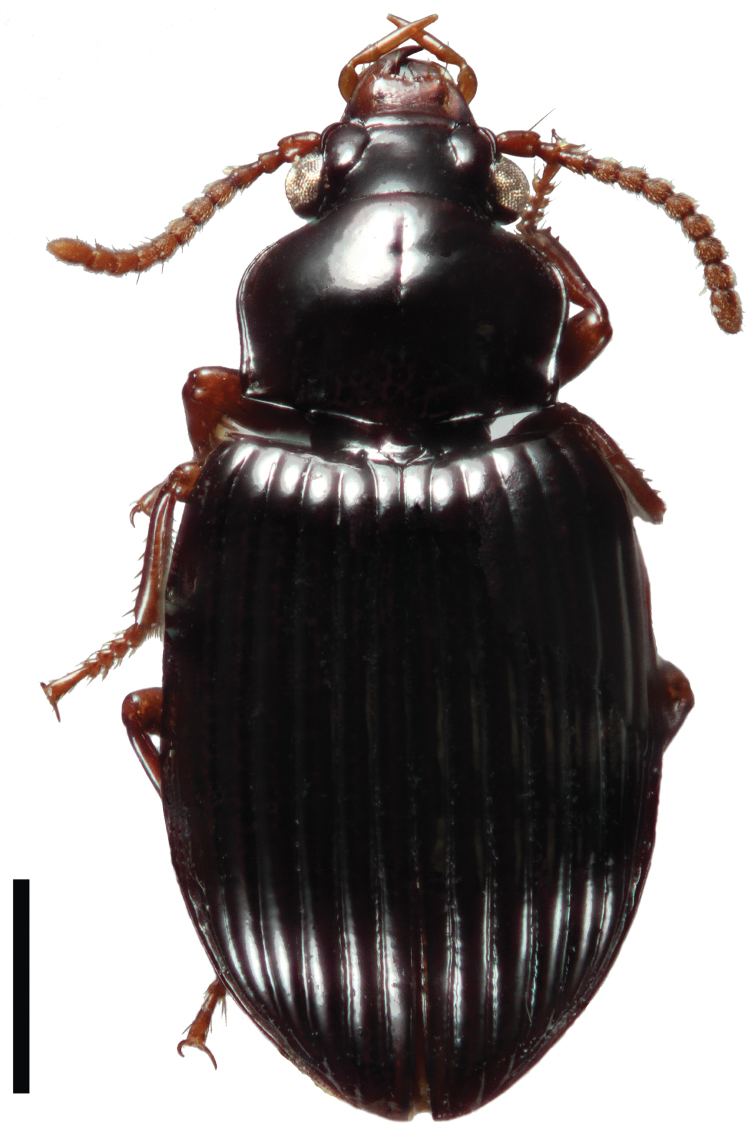
Dorsal habitus image of *Cratocerus sinesetosus* Grzymala & Will, sp. n. Scale bar = 1.0 mm.

Figs 3 , 9c , 10a , 11a , 12a

#### Type locality.

French Guiana, Region de Saul, Commune de Saul, coordinates 3.6223N, 53.2159W.

#### Type material.

HOLOTYPE [male, held in trust at NMNH until French Guiana opens its planned Natural History Museum] labeled: “FRENCH GUYANA: Region de / Saul, Commune de Saul, / Belvedere de Saul / 3.6223°N, 53.2159°W, 283–325 m / 22 Mar. 2011, flight intercept trap / S. Brule, P.H. Dalens & E. Poirier // ADP / 130028 // UC Berkeley / EMEC / 654859”.

**Paratypes.** 86 total. 2 males, with same label data as holotype (EMEC654881, EMEC654882); 2 males and 2 females, labeled: “FRENCH GUYANA: Region de Saul, Commune de Saul, Belvedere de Saul 3.6223°N, 53.2159°W, 283–325 m 30 Mar. 2011, flight & poly traps S. Brule, P.H. Dalens & E. Poirier” (EMEC654877, EMEC654878, EMEC654879, EMEC654880); 1 male, labeled: “FRENCH GUYANA: Region de Saul, Commune de Saul, Belvedere de Saul 3.6223°N, 53.2159°W, 283–325 m 14 Mar. 2011, flight intercept trap S. Brule, P.H. Dalens & E. Poirier” (EMEC654876); 1 male and 1 female, labeled: “FRENCH GUYANA: Region de Saul, Commune de Saul, Belvedere de Saul 3.6223°N, 53.2159 W°, 283–325 m 01 Mar. 2011, flight intercept trap V4 S. Brule, P.H. Dalens & E. Poirier” (EMEC654874, EMEC654875); 2 females, labeled: “FRENCH GUYANA: Region de Saul, Commune de Saul, Belvedere de Saul 3.6223°N, 53.2159°W, 283–325 m 28 Feb. 2011, flight intercept trap S. Brule, P.H. Dalens & E. Poirier” (EMEC654872, EMEC654873); 1 male, labeled: “FRENCH GUYANA: Region de Saul, Commune de Saul, Belvedere de Saul 3.6223°N, 53.2159°W, 283–325 m 23 Feb. 2011, flight intercept trap S. Brule, P.H. Dalens & E. Poirier” (EMEC654871); 1 female, labeled: “FRENCH GUYANA: Region de Saul, Commune de Saul, Belvedere de Saul 3.6223°N, 53.2159°W, 283–325 m 14 Feb. 2011, flight intercept trap V6 S. Brule, P.H. Dalens & E. Poirier” (EMEC654870); 1 female, labeled: “FRENCH GUYANA: Region de Saul, Commune de Saul, Belvedere de Saul 3.6223°N, 53.2159°W, 283–325 m 23 Jun. 2011, flight intercept trap V6 S. Brule, P.H. Dalens & E. Poirier” (EMEC654869); 2 females, labeled: “FRENCH GUYANA: Region de Saul, Commune de Saul, Belvedere de Saul 3.6223°N, 53.2159°W, 283–325 m 21 Jun. 2011, flight & poly trap S. Brule, P.H. Dalens & E. Poirier” (EMEC654867, EMEC654868); 1 male, labeled: “FRENCH GUYANA: Region de Saul, Commune de Saul, Belvedere de Saul 3.6223°N, 53.2159°W, 283–325 m 07 Jul. 2011, flight intercept trap, V1 S. Brule, P.H. Dalens & E. Poirier” (EMEC654866); 1 male, labeled: “FRENCH GUYANA: Region de Saul, Commune de Saul, Belvedere de Saul 3.6223°N, 53.2159°W, 283–325 m 11 Aug. 2011, flight intercept trap, V2 S. Brule, P.H. Dalens & E. Poirier” (EMEC654865); 1 female, labeled: “FRENCH GUYANA: Region de Saul, Commune de Saul, Belvedere de Saul 3.6223°N, 53.2159°W, 283–325 m 04 Aug. 2011, flight intercept trap, V1 S. Brule, P.H. Dalens & E. Poirier” (EMEC654864); 1 female, labeled: “FRENCH GUYANA: Region de Saul, Commune de Saul, Belvedere de Saul 3.6223°N, 53.2159°W, 283–325 m 14 Apr. 2011, flight intercept trap, V2 S. Brule, P.H. Dalens & E. Poirier” (EMEC654863); 1 male and 2 females, labeled: “FRENCH GUYANA: Region de Saul, Commune de Saul, Belvedere de Saul 3.6223°N, 53.2159°W, 283–325 m 07 Apr. 2011, flight intercept trap, V2 S. Brule, P.H. Dalens & E. Poirier” (EMEC654860, EMEC654861, EMEC654862); 5 males and 1 female, labeled: “FRENCH GUYANA: Region de Saul, Commune de Saul, Belvedere de Saul 3.6223°N, 53.2159°W, 283–325 meters 20 Dec. 2010, flight intercept trap, V4 S. Brule, P.H. Dalens & E. Poirier” (EMEC207942, EMEC207944, EMEC207945, EMEC207946, EMEC207948, EMEC207943); 1 male and 1 female, labeled: “FRENCH GUYANA: Region de Saul, Commune de Saul, Belvedere de Saul 3.6223°N, 53.2159°W, 283–325 meters 20 Dec. 2010, flight intercept trap, VPV S. Brule, P.H. Dalens & E. Poirier” (EMEC207949, EMEC207950); 1 male, labeled: “FRENCH GUYANA: Region de Saul, Commune de Saul, Belvedere de Saul 3.6223°N, 53.2159°W, 283–325 meters 20 Dec. 2010, flight intercept trap, V2 S. Brule, P.H. Dalens & E. Poirier” (EMEC207928); 1 male and 1 female, labeled: “FRENCH GUYANA: Region de Saul, Commune de Saul, Belvedere de Saul 3.6223°N, 53.2159°W, 283–325 meters 10 Dec. 2010, flight intercept trap, VPV S. Brule, P.H. Dalens & E. Poirier” (EMEC207924, EMEC207925); 4 males and 3 females, labeled: “FRENCH GUYANA: Region de Saul, Commune de Saul, Belvedere de Saul 3.6223°N, 53.2159°W, 283–325 meters 24 Jan. 2011, flight intercept trap, VPV S. Brule, P.H. Dalens & E. Poirier” (EMEC207933, EMEC207934, EMEC207935, EMEC207938, EMEC207931, EMEC207932, EMEC207936); 1 male and 1 female, labeled: “FRENCH GUYANA: Region de Saul, Commune de Saul, Belvedere de Saul 3.6223°N, 53.2159°W, 283–325 meters 17 Jan. 2011, flight intercept trap, VPV S. Brule, P.H. Dalens & E. Poirier” (EMEC207929, EMEC207927); 1 female, labeled: “FRENCH GUYANA: Region de Saul, Commune de Saul, Belvedere de Saul 3.6223°N, 53.2159°W, 283–325 meters 17 Jan. 2011, flight intercept trap, V2 S. Brule, P.H. Dalens & E. Poirier” (EMEC207939); 1 male, labeled: “FRENCH GUYANA: Region de Saul, Commune de Saul, Belvedere de Saul 3.6223°N, 53.2159°W, 283–325 meters 11 Jan. 2011, flight intercept trap, V S. Brule, P.H. Dalens & E. Poirier” (EMEC207930); 1 male, labeled: “FRENCH GUYANA: Region de Saul, Commune de Saul, Belvedere de Saul 3.6223°N, 53.2159°W, 283–325 meters 05 Feb. 2010, flight intercept trap, V5 S. Brule, P.H. Dalens & E. Poirier” (EMEC207926); 1 male, labeled: “FRENCH GUYANA: Region de Saul, Commune de Saul, Belvedere de Saul 3.6223°N, 53.2159°W, 283–325 meters 17 Feb. 2011, flight intercept trap, V1 S. Brule, P.H. Dalens & E. Poirier” (EMEC207940); 1 female, labeled: “FRENCH GUYANA: Region de Saul, Commune de Saul, Belvedere de Saul 3.6223°N, 53.2159°W, 283–325 meters 13 Aug. 2010, flight intercept trap, VPV S. Brule, P.H. Dalens & E. Poirier” (EMEC207941); 1 female, labeled: “FRENCH GUYANA: Region de Saul, Commune de Saul, Belvedere de Saul 3.6223°N, 53.2159°W, 283–325 meters 23 Sep. 2010, flight intercept trap, V5 S. Brule, P.H. Dalens & E. Poirier” (EMEC207947); 1 male, labeled: “FRENCH GUYANA: Region de Saul, Commune de Saul, Belvedere de Saul 3.6223°N, 53.2159°W, 283–325 meters 06 Oct. 2010, flight intercept trap, V2 S. Brule, P.H. Dalens & E. Poirier” (EMEC207937); 1 male and 3 females, labeled: “FRENCH GUYANA: Nouragues - Saut Parare, Commune de Regina 4.0378°N, 057.6725°W, 51m. 30 Nov. 2009, flight intercept trap S. Brule, P.H. Dalens & E. Poirier” (EMEC207904, EMEC207900, EMEC207902, EMEC207903); 1 male and 1 female, labeled: “FRENCH GUYANA: Nouragues - Saut Parare, Commune de Regina 4.0378°N, 057.725°W, 51m. 11 Dec. 2009, flight intercept trap S. Brule, P.H. Dalens & E. Poirier” (EMEC207910, EMEC207909); 1 male, labeled: “FRENCH GUYANA: Nouragues - Saut Parare, Commune de Regina 4.0378°N, 057.725°W, 51m. 28 Jan. 2010, flight intercept trap S. Brule, P.H. Dalens & E. Poirier” (EMEC207911); 2 females, labeled: “FRENCH GUYANA: Nouragues - Sant Parare, Commune de Regina 4.0378°N, 057.6725°W, 51 m. 27 Mar. 2010, flight intercept trap S. Brule, P.H. Dalens & E. Poirier” (EMEC207922, EMEC207923); 1 male, labeled: “FRENCH GUYANA: Nouragues - Saut Parare, Commune de Regina 4.0378°N, 057.725°W, 51m. 09 Apr. 2010, flight intercept trap S. Brule, P.H. Dalens & E. Poirier” (EMEC207912); 1 male and 1 female, labeled: “FRENCH GUYANA: Nouragues - Saut Parare, Commune de Regina 4.02°N, 52.41°W, 51 meters 20 Apr. 2010, flight intercept trap S. Brule, P.H. Dalens & E. Poirier” (EMEC207917, EMEC207918); 2 males, labeled: “FRENCH GUYANA: Nouragues - Saut Parare, Commune de Regina 4.02°N, 52.41°W, 51 meters 26 Apr. 2010, flight intercept trap S. Brule, P.H. Dalens & E. Poirier” (EMEC207915, EMEC207920); 2 males, labeled: “FRENCH GUYANA: Nouragues - Saut Parare, Commune de Regina 4.02°N, 52.41°W, 51m. 16 May 2010, flight intercept trap S. Brule, P.H. Dalens & E. Poirier” (EMEC207913, EMEC207919); 1 male, labeled: “FRENCH GUYANA: Nouragues - Saut Parare, Commune de Regina 4.02°N, 52.41°W, 51m. 15 Jun. 2010, flight intercept trap S. Brule, P.H. Dalens & E. Poirier” (EMEC207914); 1 female, labeled: “FRENCH GUYANA: Nouragues - Saut Parare, Commune de Regina 4.02°N, 52.41°W, 51 meters 04 Jul. 2010, flight intercept trap S. Brule, P.H. Dalens & E. Poirier” (EMEC207916); 1 male, labeled: “FRENCH GUYANA: Nouragues - Saut Parare, Commune de Regina 4.0378°N, 057.6725°W, 51m. 20 Oct. 2009, flight intercept trap S. Brule, P.H. Dalens & E. Poirier” (EMEC207905); 1 male, labeled: “FRENCH GUYANA: Reserve Naturelle des Nouragues Inselberg, Petit Plateua, Commune de Regina 4°05'N, 52°41'W, 05 Jun. 2010, flight intercept trap S. Brule, P.H. Dalens & E. Poirier” (EMEC207951); 1 male, labeled: “FRENCH GUYANA: Mount Itoupe 570 m., 03°01'19"N, 053°05'03"W, 17 Mar. 2010, flight intercept trap S. Brule, P.H. Dalens & E. Poirier” (EMEC207907); 1 male, labeled: “FRENCH GUYANA: Mount Itoupe 600 m., 03°01'19"N, 053°05'03"W, 24 Mar. 2010, flight intercept trap S. Brule, P.H. Dalens & E. Poirier” (EMEC207906); 1 male, labeled: “FRENCH GUYANA: Montagne des Chevaux Commune de Roura, RN2 PK22 4.7127°N, 52.3966°W, 90 m. 19 Dec. 2009, flight intercept trap S. Brule, P.H. Dalens & E. Poirier” (EMEC207908); 1 male, labeled: “FRENCH GUYANA: Montagne des Chevaux Commune de Roura, RN2 PK22 4.7127°N, 52.3966°W, 900 m. 26 Dec. 2010, flight intercept trap S. Brule, P.H. Dalens & E. Poirier” (EMEC207952); 1 male, labeled: “FRENCH GUYANA: Montagne des Chevaux Commune de Roura, RN2 PK22 4.7127°N, 52.3966°W, 90 m. 12 Nov. 2011, flight intercept trap S. Brule, P.H. Dalens & E. Poirier” (EMEC654883); 1 male, labeled: “FRENCH GUYANA: Montagne des Chevaux Commune de Roura, RN2 PK22 4.7127°N, 52.3966°W, 90 meters 18 Dec. 2011, flight intercept trap S. Brule, P.H. Dalens & E. Poirier” (EMEC654902); 1 male and 1 female, labeled: “FRENCH GUYANA: Montagne des Chevaux Commune de Roura, RN2 PK22 4.7127°N, 52.3966°W, 90 meters 07 Jan. 2012, flight intercept trap S. Brule, P.H. Dalens & E. Poirier” (EMEC654903, EMEC654904); 1 female, labeled: “FRENCH GUYANA: Montagne des Chevaux Commune de Roura, RN2 PK22 4.7127°N, 52.3966°W, 90 meters 03 Jan. 2012, flight intercept trap S. Brule, P.H. Dalens & E. Poirier” (EMEC654905); 1 male, labeled: “FRENCH GUYANA: Region de Saul Commune de Saul Belvedere de Saul 3.6223°N, 53.2159°W 283–325 meters 14 Aug. 2011 point of vue S. Brule P.H. Dalens & E. Poirier” (EMEC 654906); 2 males and 1 female, labeled: “PERU: MADRE DE DIOS Tambopata Rex. Zone, 290m 26 Feb 1982 T.L. Erwin 12°50'S, 069°17'W, under bark on rotten tree” (EMEC207953, EMEC207954, EMEC207955); 2 females, labeled: “PERU Madre de Dios Rio Tambopata Res. 30km (air) sw Pto. Maldonato, 290m 12°50'S, 069°20'W, Under bark P 22–26.ii 1982 N. E. Stork B. M. 1982–183” (EMEC207956, EMEC207957); 1 male, labeled: “Ecuador: Napo Limoncocha 10 June 1977 W.E. Steiner” (EMEC207959). All paratype specimens deposited in NMNH except specimens EMEC654881, EMEC654872 deposited in MNHN, specimens EMEC207948, EMEC207950 deposited in EMEC, and specimen EMEC207957 deposited in Museo de Historia Natural, Lima, Peru (UNMSM). When the Natural History Museum in French Guiana has completed construction several specimens currently held in NMNH will be transferred there.

#### Diagnosis.

This species is distinguished from other members of *Cratocerus* by the absence of setae on the apicolateral bead of the pronotum. This species most closely resembles *Cratocerus sulcatus* and *Cratocerus multisetosus* in overall body shape and coloration, but is easily separated by the absence of apicolateral pronotal setae, whereas there is one pair of apicolateral setae present in *Cratocerus sulcatus* and two or three pairs present on the pronota of *Cratocerus multisetosus*.

#### Description.

Size average for the genus, ABL 5.4–6.1 mm, TW 2.3–2.7 mm. *Color*. Head, prothorax, and elytra piceous. Labrum, mandibles, mentum, and submentum brunneopiceous. Antennae, legs, and remaining mouthparts testaceous. *Head*. HL 0.7–0.9 mm, HW 0.8–1.0 mm. One supraorbital seta present over each eye. Apices of mandibles slightly curved, at less than 45 degree angle to mandibular midline. Apical maxillary palpomere length 5 × width at base. Mentum with triangular lateral lobes. *Thorax*. Pronotum (Fig. 9c) with distinct median impression, one distinct basal fovea laterad; no seta located on apicolateral pronotal bead; one posterior seta present on posterior bead near posterior angle; pronotal anterior margin convex; pronotal width 1.1–1.3 × pronotal length. Elytral length 1.3–1.5 × combined elytral width. *Legs*. Protibia with three distinct, stout spines confined to distal half of lateral margin; distance between spines 1 and 2 slightly less than distance between spines 2 and 3 (Fig. 10a). *Genitalia, male*. Median lobe curved sharply, between forty-five and ninety degrees, with respect to basal 1/3, remaining 2/3 with continuing curvature; not expanded apically. Left paramere complexly curved into an “S” shape with basal projection (Figure 11a). *Genitalia, female*. Spermatheca with 2/3 length thin, gradually expanding approximately 1/3 below apex, ended with broad bulb (Fig. 12a).

#### Etymology.

The specific epithet refers to the absence of any anterior marginal setae on the pronotum of this species. The name is formed by combining the Latin *sine* (without) and *setosus* (setose); adjective.

#### Distribution.

This species is distributed (Fig. 13) throughout French Guiana, Peru, and Ecuador.

#### Ecology.

Specimens have been collected from underneath the bark of rotting trees during the month of February and from flight intercept traps throughout the year.

### Cratocerus multisetosus

Taxon classificationAnimaliaColeopteraCarabidae

Grzymala & Will sp. n.

http://zoobank.org/AA11EA3B-19C2-46AC-A262-315C899447E2

**Figure 4.**
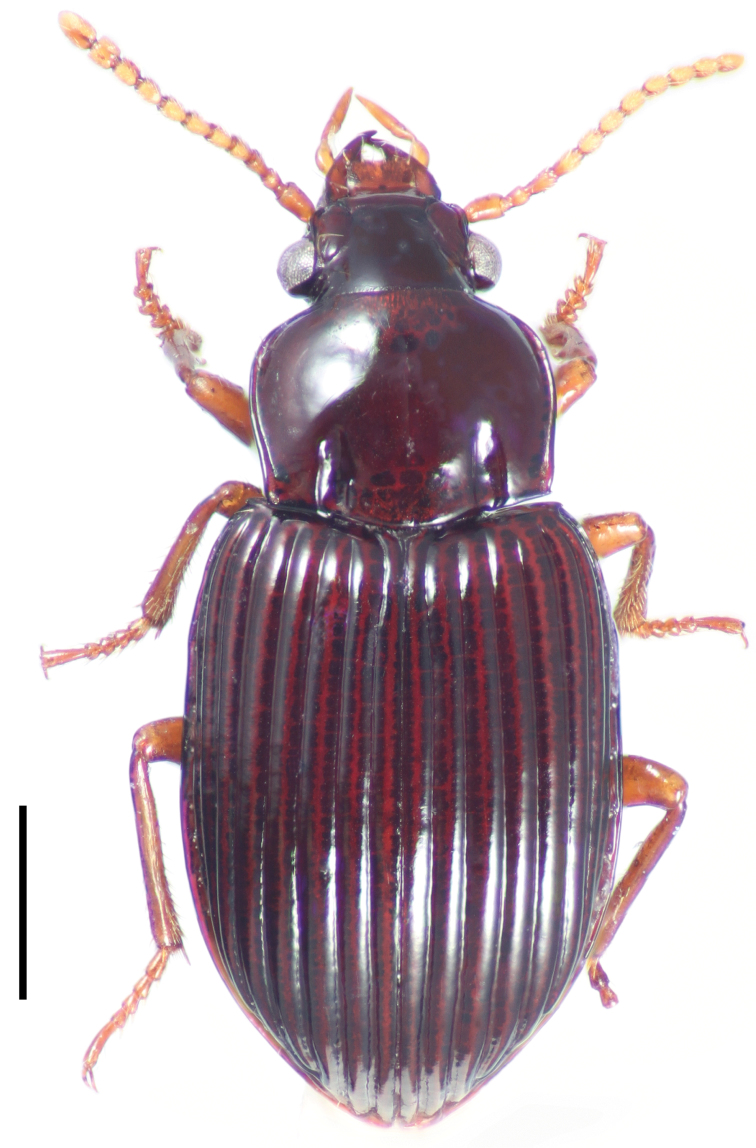
Dorsal habitus image of *Cratocerus multisetosus* Grzymala & Will, sp. n. Scale bar = 1.0 mm.

Figs 4 , 9d , 10b , 11b

#### Type locality.

Costa Rica, Heredia Province, La Selva, 3 km S Puerto Viejo, coordinates 10.42N, 84.00W.

#### Type material.

HOLOTYPE [male, deposited in INBio] labeled: “COSTA RICA: Heredia, La / Selva (3 km S Puerto Viejo, / 10°25'N, 84°0'W); 80m; / 14 June 1996; R.S. Hanley / fogged from downed logs- / Arboretum; #RSHCR052// Cratocerus / sp. 2 // ADP / 105074 // U.C. Berkeley / EMEC 207,970”

**Paratypes.** 19 total; 1 male and 1 female, labeled: “COSTA RICA: Heredia, La Selva (3 km S Puerto Viejo, 10°25'N, 84°0'W); 80m; 14 June 1996; R.S. Hanley fogged from downed logs- Arboretum; #RSHCR052” (EMEC207971, EMEC207972); 1 male, labeled: “Sector Cocori, 30 Km N. de Cariari, Finca E. Rojas, Prov. Limon, COSTA RICA. 100m. Mar 1994. E. Rojas L N 286000_567500 # 2790” (EMEC207974); 1 female, labeled: “Sector Cocori, 30 km al N. e Cariari, Prov Limon, COSTA RICA, 100 m. Feb 1994, E. Rojas, A. A. Solis, L N 286000_567500 #2639” (EMEC207973); 1 male, labeled: “Est. Pitilla, 9 km S. Sta. Cecilia, P. N. Guanacaste, Prov. Guana, COSTA RICA. 700 m. 3–17 Dic 1993, C. Moraga, L N 330200_380200 #2545” (EMEC207975); 1 male, labeled: “Estac. Pitilla, 700m, 9km S Santa Cecilia, Guanac. Pr. COSTA RICA. Dic 1989 C. Moraga & P. Rios 330200” (EMEC207981); 1 male, labeled: “Est. Queb. Bonita, 50m, Res. Biol. Carara, Prov. Punt. COSTA RICA, E. Bello, Jun 1990. L_N_194500, 469850” (EMEC207983); 1 male, labeled: “Estacion Pitilla 9 km. S. de Santa Cecilia, Prov. Guana, COSTA RICA. 700m. ENE 1995. P. Rios, L_N_329950_380450 #4358” (EMEC207982); 1 female, labeled: “COSTA RICA, Prov. Puntarenas, Golfito, Reserva Ftal Golfo Dulce, Estacion Agujas. 250-350m. 13 JUL 1999. A. Azofeifa de Luz L_S_276750_526550 #53819” (EMEC207976); 1 female, labeled: “COSTA RICA. Prov. Limon. Res Biol Hitoy Cerere. Est. Hitoy Cerere, Send Espavel. 360m. 25 MAY 1998. W. Arana. Manual (red, libre) L S 401500 570200 #56427” (EMEC207977); 1 male, labeled: “COSTA RICA. Prov. Puntarenas, P.N. Corcovado, Est. Sirena, Send. Espavel, 0-100m, 19 MAR 2001, A. Azofeifa, Entrocos L_S_269900_507750 #64447” (EMEC207980); 1 male, labeled: “COSTA RICA Puntarenas Prov. Osa Peninsula 2.5 mi. SW. Rington 08°42'N, 83°29'W, III-1 to 7- 1967 OTS Adv. Zoo. Course” (EMEC207985); 1 female, labeled: “CR: Punta: Corocovado N.P., Sirena Station Ollas Trail. On/under bark, downed log elev. 10m 30-V-92” (EMEC207978); 1 male, labeled: “Turrialba Costa Rica 19 June 1951 OL Cartwright” (EMEC207984); 1 female, labeled: “74k29 slide 148 Turrialba Costa Rica 6 June 1951 OL Cartwright” (EMEC207979); 1 male, labeled: “COSTA RICA F NEVERMANN Salvadora Farm Parismina 5-10.IX.1930 in trocknem” (EMEC207987); 1 male, labeled: “COSTA RICA F NEVERMANN Salvadora Farm Parismina Fluss 19–31.XII.1930 in morschen” (EMEC207988); 1 male, labeled: “COSTA RICA F NEVERMANN 5 II 28 HAMBURG FARM REVENTAZON / EBENE LIMON in morschen” (EMEC207989); 1 male, labeled: “Panama: Panama Pr. Cerro Campana, 850m 8°40'N, 79°56'W, 12 Sept. ‘70 H Stockwell” (EMEC207986). All deposited in INBio except EMEC207978, EMEC207984, EMEC207979, EMEC207987, EMEC207988, EMEC207989, and EMEC207986 deposited in NMNH and EMEC207982, EMEC207976 deposited in EMEC.

#### Diagnosis.

Distinguishable from similar looking species such as *Cratocerus sulcatus* and *Cratocerus sinesetosus*, by the additional setae on the pronotal margin (Fig. 9d) and moderately convex elytral intervals.

#### Description.

Size average for the genus, ABL 5.2–5.8 mm, TW 2.5–2.7 mm. *Color*. Head and prothorax brunneopiceous, elytra and abdominal sternites brunneous, legs, antennae, and mouthparts rufotestaceous. *Head*. HL 0.7–0.9 mm, HW 0.7–0.9 mm. One supraorbital seta present over each eye. Apices of mandibles slightly curved, at less than 45 degree angle to mandibular midline. Apical maxillary palpomere length 5 × width at base. Mentum with triangular lateral lobes. *Thorax*. Pronotum (Fig. 9d) with distinct median impression, one distinct basal fovea laterad; two to three lateral setae located on apicolateral pronotal bead; one posterior seta present on posterior bead near posterior angle; pronotal anterior margin convex; pronotal width 1.2–1.3 × pronotal length. Elytral length 1.4–1.5 × combined elytral width. *Legs*. Protibia with three distinct, stout spines confined to distal half of lateral margin; distance between spines 1 and 2 slightly less than distance between spines 2 and 3 (Fig. 10b). *Genitalia, male*. Median lobe curved sharply, between forty-five and ninety degrees, with respect to basal 1/3, remaining 2/3 with continuing curvature; not expanded apically; length average for genus. Left paramere complexly curved into an “S” shape with basal projection (Figure 11b). *Genitalia, female*. Spermatheca with 2/3 length thin, gradually expanding approximately 1/3 below apex, ended with broad bulb, similar to *Cratocerus sinesetosus* (Fig. 12a).

#### Etymology.

The specific epithet refers to the two or three anterior marginal setae on each side of the pronotum found in this species. The name is formed by combining the Latin *multi* (many) and *setosus* (setose); adjective.

#### Distribution.

This species is distributed (Fig. 13) throughout Costa Rica and has also been collected in Panama.

### Cratocerus tanyae

Taxon classificationAnimaliaColeopteraCarabidae

Grzymala & Will sp. n.

http://zoobank.org/D830BA01-460D-4E9B-8848-0FF697B94BD4

**Figure 5.**
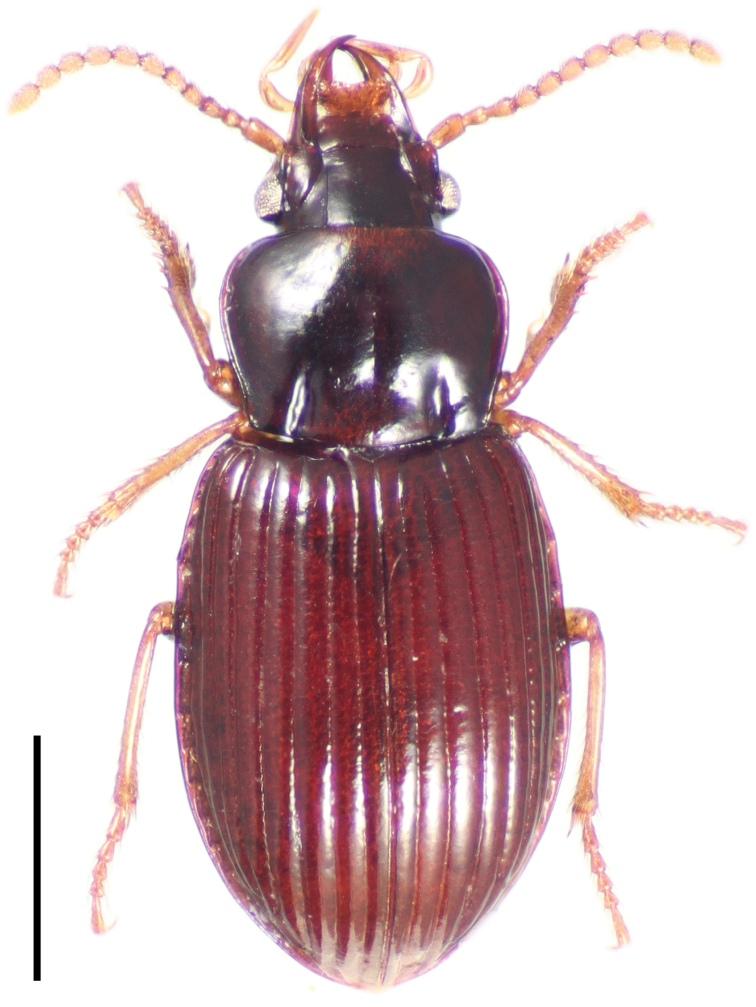
Dorsal habitus image of *Cratocerus tanyae* Grzymala & Will, sp. n. Scale bar = 1.0 mm.

Figs 5 , 9d , 10c , 11d

#### Type locality.

Costa Rica, Limon Province, Reserva Biológica Hitoy-Cerere, Sendero Bobocara. Estimated coordinates 9.67N, 83.03W.

#### Type material.

HOLOTYPE [male, deposited in INBio] labeled: “COSTA RICA. Prov. Limon Reserva / Biol Hitoy Cerere, Send Bobocara. / 200m. 13 ABR 2000. W. Arana / Mantillo L_N_184700_641200 #56335 // INB0003076608 / INBIOCRI COSTA RICA // U.C. Berkeley / EMEC 207,842”.

**Paratypes.** 19 total; 1 female, labeled: “Sector Cerro Cocori, Finca de E. Rojas, Prov. Limon, COSTA RICA, 150m. SET 1994, E. Rojas, L_N_286000_567500 #3218” (EMEC207845); 1 female, labeled: “COSTA RICA. Prov. Limon, R.B. Hitoy Cerere, A.C.L.A.C. Estac. Hitoy Cerere, Send, Catarata, 200m, 30 SEP 2000. W. Arana, Mantillo con Sopa L_N_184550_643250 #63428” (EMEC207999); 1 male and 1 female, labeled: “Est. Quebrada Bonita, Prov. Punta, COSTA RICA. 50m. Nov 1994 R. Guzman, L N 194500_469850 #3258” (EMEC207990, EMEC207994); 1 male, labeled: “Est. Q. Bonita, Prov. Punta, COSTA RICA. 50m. Jun 1993. R. Guzman, L N 194500_469850 #2202” (EMEC207991); 1 male and 1 female, labeled: “Est. Queb. Bonita, 50m, Res. Biol. Carara, Prov. Puntarenas, Costa Rica Dic 1992, R. Guzman L_N 194500, 469850” (EMEC207992, EMEC207993); 1 male, labeled: “COSTA RICA. Prov. Limon, Est. R.B. Hitoy Cerere, La poza de las chichis, 140m. 18-29 JUN 1998. E. Rojas, Tp. Mantillo L_N_184176_643226 #50751” (EMEC207841); 1 female, labeled: “COSTA RICA. Prov. Puntarenas, P.N. Corcovado, Est. Sirena, Send, Espavel, 0-100m, 20 MAR 2001, A. Azofeifa, Mantillo L_S_269900_507750 #64457” (EMEC207846); 1 female, labeled: “COSTA RICA: Prov. Limon. R.B. Hitoy Cerere Send. a Espavel. 200m. 22 ENE 2003. W. Arana. Tp de Mantillo I. N. 184700 644200 #72874” (EMEC654899); 2 males and 1 female, labeled: “COSTA RICA: Puntarenas Reserva Biol. Carana 09°47'N, 84°36'W, 30m 23 July 1985, J. Longino #534-5, ex., leaf litter” (EMEC207995, EMEC207996, EMEC207997); 1 female, labeled: “COSTA RICA. Prov. Limon, Res Biol Hitoy Cerere, Est Hitoy Cerere, Send Bobocara. 300m. 11 FEB 2000. W. Arana Mantillo L_N_184250_641800 #56325” (EMEC207840); 1 female, labeled: “COSTA RICA. Prov. Limon, Central, Reserva Biol Hitoy Cerere, Est Hitoy Cerere. Send Tepezquintle 140m. 9-11 SET 1999. W. Arana Mantillo L_N_184250_643200 #56886” (EMEC207844); 1 female, labeled: “COSTA RICA. Prov. Limon R.B. Hitoy Cerere Sendero Tepezcuintle 0–100m.14 ENE 2003. W. Arana Tp. de Mantillo. I.N. 184200 643200 #72872” (EMEC654901); 1 female, labeled: “GUAT. Isabal Las Escobas 12.XI.1986 M. Sharkey” (EMEC654885); 1 male, labeled: “Ayutla, Guat. VIII-19-1947 B. Malkin” (EMEC654884); 1 male, labeled: “MEX. Chis., Palenque 28.VII.1983, rain forests, S. & J. Peck ultraviolet light” (EMEC654886). All specimens deposited in INBio except EMEC207991, EMEC207995, EMEC207997, EMEC207844 deposited in NMNH and EMEC207995, EMEC207846 deposited in EMEC.

#### Diagnosis.

This species most closely resembles *Cratocerus indupalmensis* and can be distinguished by the smaller size, differently shaped pronotum (Fig. 9e, f), and the differently sized and shaped median lobe of the male genitalia (Fig. 11d, e).

#### Description.

Size small for the genus, ABL 4.1–4.7 mm, TW 1.8–2.1 mm. *Color*. Head and prothorax brunneopiceous; elytra, abdominal sternites, and mandibles brunneous; legs, antennae, and remaining mouthparts rufotestaceous. *Head*. HL 0.5–0.7 mm, HW 0.6–0.8 mm. One supraorbital seta present over each eye. Apices of mandibles slightly curved, at less than 45 degree angle to mandibular midline. Apical maxillary palpomere length 6 × width at base. Mentum with triangular lateral lobes. *Thorax*. Pronotum (Fig. 9d) with distinct median impression, one distinct basal fovea laterad; one lateral seta located on apicolateral pronotal bead; one posterior seta present on posterior bead and projecting over posterior angle; pronotal anterior margin convex, greatest width just anterad of center; pronotal width 1.3–1.4 × pronotal length. Elytral length 1.3–1.5 × combined elytral width. *Legs*. Protibia with three distinct, stout spines confined to distal half of lateral margin; distance between spines 1 and 2 approximately half of distance between spines 2 and 3 (Fig. 10c). *Genitalia, male*. Median lobe curved sharply, between forty-five and ninety degrees, with respect to basal 1/3, remaining 2/3 with continuing curvature; not expanded apically; length short for genus. Left paramere simply curved (Figure 11d). *Genitalia, female*. Spermatheca with entire length thin, not gradually expanding towards apex, form similar to spermatheca of *Cratocerus kavanaughi* (Fig. 12c), but much longer (2× length) and relatively narrower (1/2× width).

#### Etymology.

This species is named in recognition of Tanya Shahjanian. As an undergraduate student at the University of California, Berkeley, Tanya participated in an insect art and culture project competition and was selected by the panel of judges as the winner with a sculpture piece called “*It's an insect world*”. As a prize this fine species of beetle is named for her.

#### Distribution.

This species is distributed (Fig. 13) from Mexico through the Central American countries of Guatemala and Costa Rica.

#### Ecology.

Specimens have been collected from underneath decaying wood and at ultraviolet light traps from July to November.

### Cratocerus indupalmensis

Taxon classificationAnimaliaColeopteraCarabidae

Grzymala & Will sp. n.

http://zoobank.org/BFD57836-FE56-474C-9A96-C47A6075D3E3

**Figure 6.**
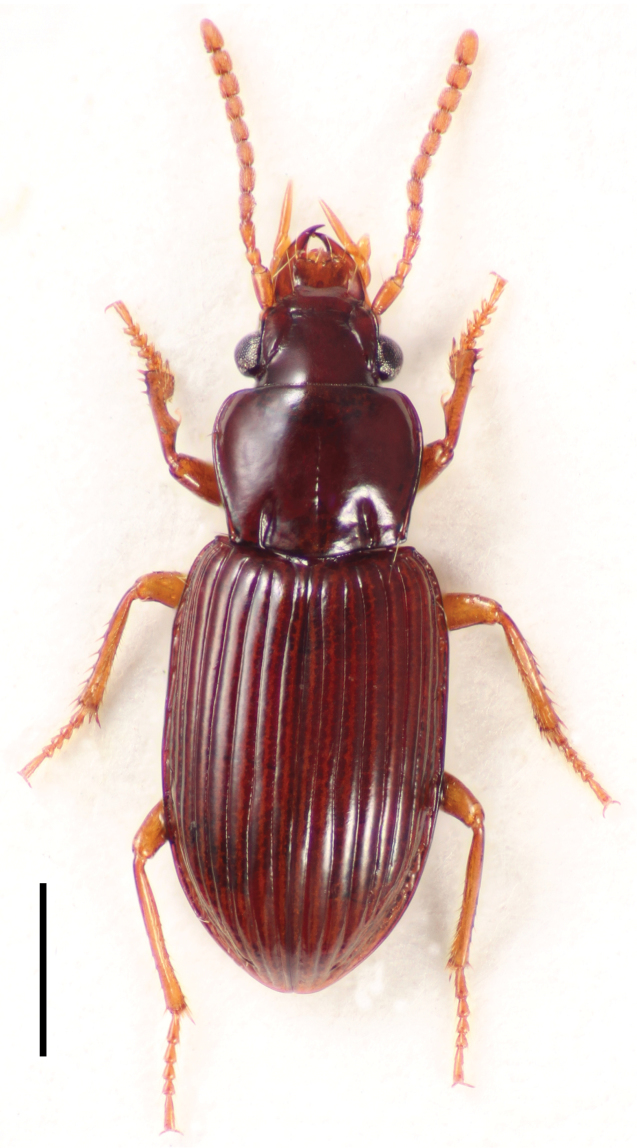
Dorsal habitus image of *Cratocerus indupalmensis* Grzymala & Will, sp. n. Scale bar = 1.0 mm.

Figs 6 , 9f , 10d , 11e

#### Type locality.

Costa Rica, Puntarenas Province, Canton de Osa, Peninsula de Osa, Rancho Quemado. Estimated coordinates 8.70N, 83.55W.

#### Type material.

HOLOTYPE [male, deposited in INBio] labeled: “Rancho Quemado, Penin- / sula de Osa, 200m. Prov, / Punt., COSTA RICA, / F. Quesada, Dic 1991, / L_S_292500, 511000 // COSTA RICA INBIO / CRI000 / 539764 // *Cratocerus* / *parallelus* // U.C. Berkeley / EMEC 207,998”.

**Paratypes.** 36 total. 1 male, labeled: “COSTA RICA: Limon Reventazon, Hamburg Farm 25:10:35 leg. F. Nevermann FM(HD)#35-10 in decaying wood Field Mus. Nat. Hist. 1966 A Bierig Colln. Acc. Z- 13812” (EMEC207843); 1 male and 1 female, labeled: “PANAMA. Barro Colorado Island 6-8 Dec. 1971 beneath loose bark-stump T.L. and L.J. Erwin Collectors” (EMEC654888, EEMC654887); 1 female, labeled: “EL SALVADOR Vol. Conchagua 27–29 May 1958 OL Cartwright” (EMEC654893); 1 male, labeled: “BELIZE: Toledo Dist. Blue Creek Village 18 June, 1981 W.E. Steiner Earthwatch Belize Expedition 1981 D.H. Messersmith W.E. Steiner, et. al” (EMEC654890); 1 male, labeled: “Tejupilco, Mex. Temescaltepec ca. 4000ft. 1933 B.M. 1959-100. H.E. Hinton R.L. Usinger Collectors” (EMEC654891); 1 female, labeled: “Mixco, Guat May, 1924 WM Mann” (EMEC654895); 1 female, labeled: “Toledo Dist. Brit. Hond. 10/15 ‘06 Peck F.C. Bowditch Coll.” (EMEC654894); 1 male, labeled: “ECUADOR: NAPO Cabanas / Alinahui, 11km E Pto. Misahualli 425m, 2 June 1993 01°04'00"S, 077°36'00"W, Erwin/Servat Under bark of old, dry, fallen tree, in forest south of Tena- Ahuano Rd. Lot 473” (EMEC207854); 2 males, labeled: “PERU: MADRE DE DIOS Tambopata Res. Zone, 290m 26 Feb 1982 T.L. Erwin 12°50'S, 069°17'W, Under bark on rotten tree” (EMEC207847, EMEC207848); 1 male and 1 female, labeled: “PERU: MADRE DE DIOS Pakitza 12Oct90 T.L. Erwin & M.G. Pogue Colls 12°07'S, 70°58'W, Under bark Radial 3, St. 45” (EMEC207849, EMEC207850); 1 male, labeled: “ PERU Dpto. Loreto 1 km SW Boca del Rio Samiria 04°40'29"S, 74°18'55"W, 130m. under bark 15.VIII.1991 05-91 T.L. ERWIN PERU EXP. Res. Pacaya- Samiria G.E. Ball & D. Shpeley collectors 1991” (EMEC207852); 1 female, labeled: “PERU: LORETO, Cocha Shinguito 27 May 90, 74°45'W, 05°08'S, Erwin et al colls TShs/1; under bark of rotten branch” (EMEC207853); 1 female, labeled: “PERU LORETO 1 km SW Boca del Rio Samiria Vigilante Post No. 1 130m 15 Aug 1991 04°40.5'S, 74°18.9'W, Under bark and on logs in restringa forest off Tr. Norte Lot 12 T.L. Erwin” (EMEC654889); 1 female, labeled: “FRENCH GUYANA: Region de Saul, Commune de Saul, Belvedere de Saul 3.6223N, 53.2159W, 283-325 meters 24 Jan. 2011, flight intercept trap, VPV S. Brule, P.H. Dalens & E. Poirier” (EMEC654892); 2 males, labeled: “Brasilien Nova Teutonia 27°11 B 52°23 L Fritz Plaumann IX 1948 19” (EMEC207855, EMEC207856); 1 female, labeled: “S. Paulo d’Olivenca Amazones M de Mathan Cratocerus vois bronilinni Chd” (EMEC207857); 1 female, labeled: “Rio Jan. Sulcatus Chaud Bates coll” (EMEC207858); 9 males and 6 females, labeled: “Colombia / San Alberto / Indupalma / Dro Cesar / IX-X-XI-1968” (EMEC207864, EMEC207861, EMEC207863, EMEC207866, EMEC207867, EMEC207870, EMEC207871, EMEC207872, EMEC207874, EMEC207869, EMEC207860, EMEC207862, EMEC207865, EMEC207868, EMEC207873). Specimens with unique identifier label EMEC207855, EMEC207856, EMEC207857, EMEC207858, EMEC207864, EMEC207861, EMEC207863, EMEC207866, EMEC207867, EMEC207870, EMEC207871, EMEC207872, EMEC207874, EMEC207869, EMEC207860, EMEC207862, EMEC207865, EMEC207868, EMEC207873 deposited in MNHN. All remaining specimens deposited in NMNH except EMEC207847 deposited in UNMSM, EMEC207852 deposited in University of Alberta Entomology Collection (UASM), and EMEC207848, EMEC207853 deposited in EMEC.

#### Diagnosis.

This species is most easily confused with *Cratocerus tanyae* and can generally be separated by its slightly larger overall body size and the differently shaped pronotum (Fig. 9e, f). The size and shape of the median lobe of the male genitalia distinctly diagnose these two species (Fig. 11d, e).

#### Description.

Size average for the genus, ABL 4.8–5.5 mm, TW 1.8–2.4 mm. *Color*. Head and prothorax brunneopiceous; elytra and abdominal sternites brunneous; legs, antennae, and mouthparts rufotestaceous. *Head*. HL 0.7–0.9 mm, HW 0.7–0.8 mm. One supraorbital seta present over each eye. Apices of mandibles slightly curved, at less than 45 degree angle to mandibular midline. Apical maxillary palpomere length 6 × width at base. Mentum with triangular lateral lobes. *Thorax*. Pronotum (Fig. 9f) with distinct median impression, one distinct basal fovea laterad; one lateral seta located on apicolateral pronotal bead; one posterior seta present on posterior bead and projecting over posterior angle; pronotal anterior margin convex, greatest width anterad of center; pronotal width 1.2–1.3 × pronotal length. Elytral length 1.5–1.7 × combined elytral width. *Legs*. Protibia with three distinct, stout spines confined to distal half of lateral margin; distance between spines 1 and 2 equidistant to distance between spines 2 and 3; one additional smaller spine laterad to spine 1 and one additional smaller spine anterad to spine 1, both only slightly visible with magnification of 125×, clearly visualized with scanning electron microscopy (Fig. 10d). *Genitalia, male*. Median lobe curved sharply, between forty-five and ninety degrees with respect to basal 1/3, remaining 2/3 with continuing curvature; very slight apical expansion; length average for the genus. Left paramere simply curved (Figure 11e). *Genitalia, female*. Spermatheca extremely thin, filamentous, form similar to spermatheca of *Cratocerus kavanaughi* (Fig. 12c), but much longer (2× length) and relatively narrower (1/2× width).

#### Etymology.

The specific epithet refers to a series of specimens bearing locality labels specifying “Indupalma”. This series represent the specimens that were first recognized as constituting part of this quite variable new species of *Cratocerus*. *Indupalma* is treated as an adjective.

#### Distribution.

This widespread species (Fig. 13) has been collected in Belize, Brazil, Colombia, Costa Rica, Ecuador, El Salvador, French Guiana, Guatemala, Mexico, Panama, and Peru.

#### Ecology.

This species has been collected under the bark of rotting, fallen trees.

### Cratocerus kavanaughi

Taxon classificationAnimaliaColeopteraCarabidae

Grzymala & Will sp. n.

http://zoobank.org/DD97E235-56AB-46C3-9003-9956C66D1A96

**Figure 7.**
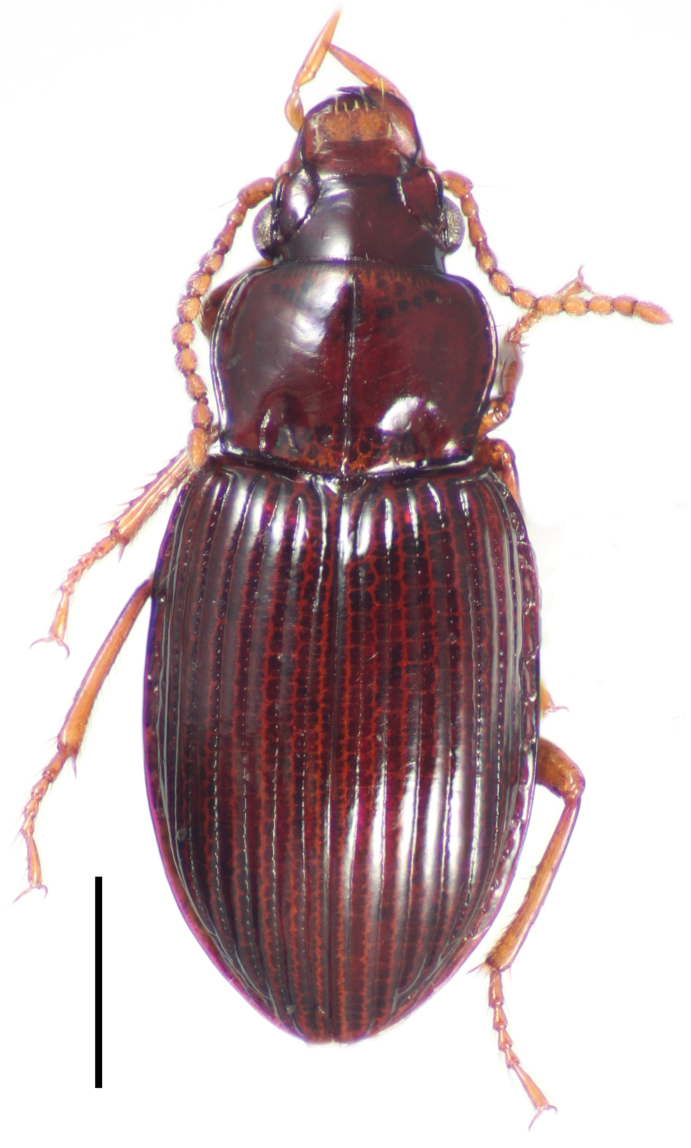
Dorsal habitus image of *Cratocerus kavanaughi* Grzymala & Will, sp. n. Scale bar = 1.0 mm.

Figs 7 , 9g , 10e , 11f , 12c

#### Type locality.

French Guiana, Nouragues Scientific Station, Pararé site, coordinates 4.02N, 52.41W.

#### Type material.

HOLOTYPE [male, deposited in NMNH] labeled: “FRENCH GUYANA: Nouragues - / Saut Parare, Commune de Regina / 4.02°N, 52.41°W, 51 meters / 26 Apr. 2010, flight intercept trap / S. Brule, P.H. Dalens & E. Poirier // ADP / 126766 // U.C. Berkeley / EMEC / 654851”.

**Paratypes.** 10 total; 1 female, labeled: “FRENCH GUYANA: Nouragues - Saut Parare, Commune de Regina 4.02°N, 52.41°W, 51 meters 20 Apr. 2010, flight intercept trap S. Brule, P.H. Dalens & E. Poirier (EMEC207921); 1 female, labeled: “FRENCH GUYANA: Nouragues - Saut Parare, Commune de Regina 4.0378°N, 057.6725°W, 51m. 30 Nov. 2009, flight intercept trap S. Brule, P.H. Dalens & E. Poirier” (EMEC207901); 1 male, labeled: “FRENCH GUYANA: Reserve Naturelle des Nouragues Inselberg, Camp, Commune de Regina, 4°05'N, 52°41'W, flight intercept trap 23 February 2011 S. Brule, P.H. Dalens & E. Poirier” (EMEC654857); 1 male, labeled: “FRENCH GUYANA: Montagne des Chevaux Commune de Roura, RN2 PK22, 4.7127°N, 52.3966°W, 90 meters 21 Dec. 2011, flight intercept trap S. Brule, P.H. Dalens & E. Poirier” (EMEC654907); 1 female, labeled: “FRENCH GUYANA: Montagne des Chevaux Commune de Roura, RN2 PK22, 4.7127°N, 52.3966°W, 900 m. 26 Dec. 2010, flight intercept trap S. Brule, P.H. Dalens & E. Poirier” (EMEC654852); 1 female, labeled: “FRENCH GUYANA: Montagne des Chevaux Commune de Roura, RN2 PK22, 4.7127°N, 52.3966°W, 90 meters 23 July 2011, SLAM Malaise trap S. Brule, P.H. Dalens & E. Poirier” (EMEC654856); 1 female, labeled: “FRENCH GUYANA: Region de Saul, Commune de Saul, Belvedere de Saul, 3.6223N, 53.2159W, 283–325 meters 24 Jan. 2011, flight intercept trap, VPV S. Brule, P.H. Dalens & E. Poirier” (EMEC654853); 1 female, labeled: “FRENCH GUYANA: Region de Saul, Commune de Saul, Belvedere de Saul 3.6223N, 53.2159W, 283–325 meters 07 Apr. 2011, flight intercept trap, V6 S. Brule, P.H. Dalens & E. Poirier” (EMEC654854); 1 male, labeled: “FRENCH GUYANA: Region de Saul, Commune de Saul, Belvedere de Saul 3.6223N, 53.2159W, 283-325 meters 20 Feb.. 2010, flight intercept trap, V5 S. Brule, P.H. Dalens & E. Poirier” (EMEC654845); 1 male, labeled: “PERU: MADRE DE DIOS Pakitza, Zone 2 07Oct90 T.L. Erwin & M.G. Pogue Colls, 12°07'S, 70°58'W, Under bark on Tr. Aguajal nr. St. 127” (EMEC207851). All specimens deposited in NMNH except EMEC654857 deposited in MNHN.

#### Diagnosis.

This species is distinguished from other members of *Cratocerus* by the strongly curved mandibles, 45 degrees in relation to the mandibular midline and the two basal pronotal fovea. This species is most easily confused with *Cratocerus culpepperi* as both have the distinctively curved mandibles, a character which does not occur in any other species within *Cratocerus*. *Cratocerus kavanaughi* is easily identified by the larger overall body size and the two pronotal basal fovea as opposed to the five found with *Cratocerus culpepperi* specimens (Fig. 9g, h).

#### Description.

Size average for the genus, ABL 4.8–5.6 mm, TW 1.9–2.4 mm. *Color*. Head and prothorax brunneopiceous; elytra, abdominal sternites, and mandibles brunneous; legs, antennae, and remaining mouthparts rufotestaceous. *Head*. HL 0.7–1.0 mm, HW 0.8–0.9 mm. One supraorbital seta present over each eye. Apices of mandibles strongly curved, apices at more than 45 degree angle to mandibular midline. Apical maxillary palpi length 4 × width at base. Mentum with triangulate lateral lobes. *Thorax*. Pronotum (Fig. 9g) with distinct median impression, one distinct basal fovea laterad; one lateral seta located on apicolateral pronotal bead; one seta present on posterior bead and projected over posterior angle; pronotal anterior margin convex, greatest width anterad of center; pronotal width 1.3–1.4 × pronotal length. Elytral length 1.4–1.6 × elytral width. *Legs*. Protibia with three small, stout spines confined to distal half of lateral margin, only visible with extreme magnification (Fig. 10e). *Genitalia, male*. Median lobe curved sharply, between forty-five and ninety degrees with respect to basal 1/3, remaining 2/3 without continuing curvature; narrowed apically; length average for species of the genus. Left paramere curved and simple (Figure 11f). *Genitalia, female*. Spermatheca with entire length equal and moderate, length average for genus; apex rounded, without broad bulb (Fig. 12c).

#### Etymology.

The specific epithet, *kavanaughi*, is a Latinized version (genitive case) of the surname of Dr. David H. Kavanaugh, an entomology curator at the California Academy of Sciences. Dr. Kavanaugh provided numerous resources and support to TLG throughout the duration of this project. This species is named in gratitude of this support.

#### Distribution.

This species is geographically distributed (Fig. 13) throughout French Guiana and Peru.

#### Ecology.

Specimens have been collected in flight intercept traps during the months of November, December, January, February, April, and July and under tree bark in October.

### Cratocerus culpepperi

Taxon classificationAnimaliaColeopteraCarabidae

Grzymala & Will sp. n.

http://zoobank.org/B4B00EFA-34CC-4DCC-83B3-40CF563F9303

**Figure 8.**
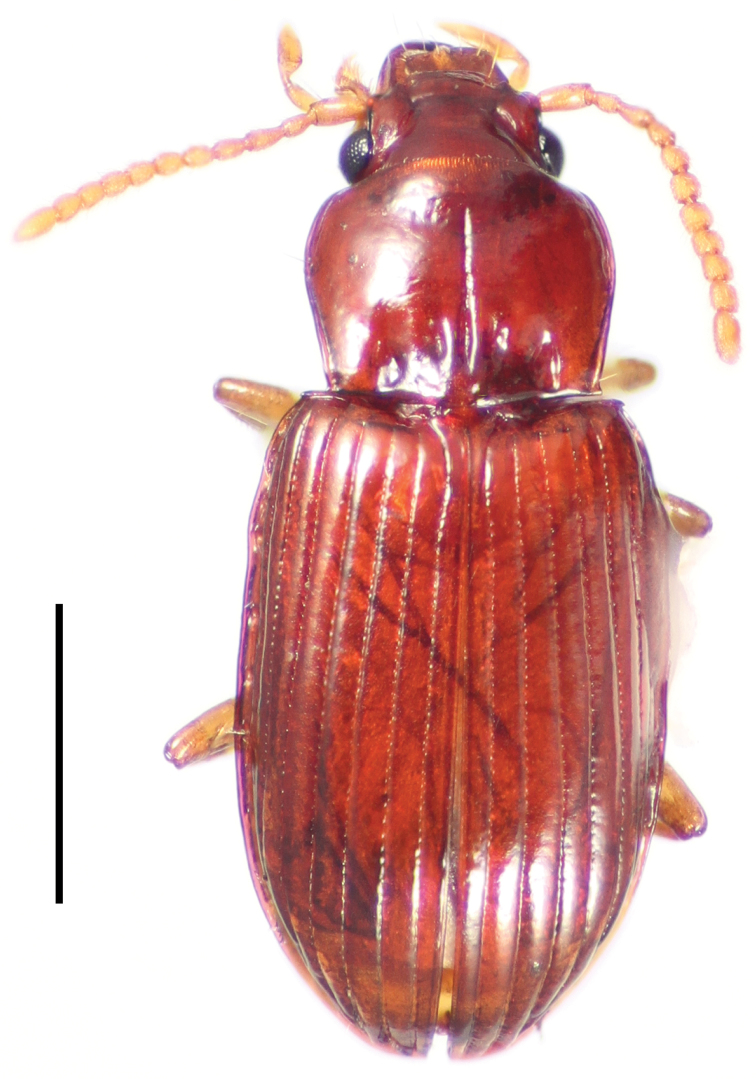
Dorsal habitus image of *Cratocerus culpepperi* Grzymala & Will, sp. n. Scale bar = 1.0 mm.

**Figure 9.**
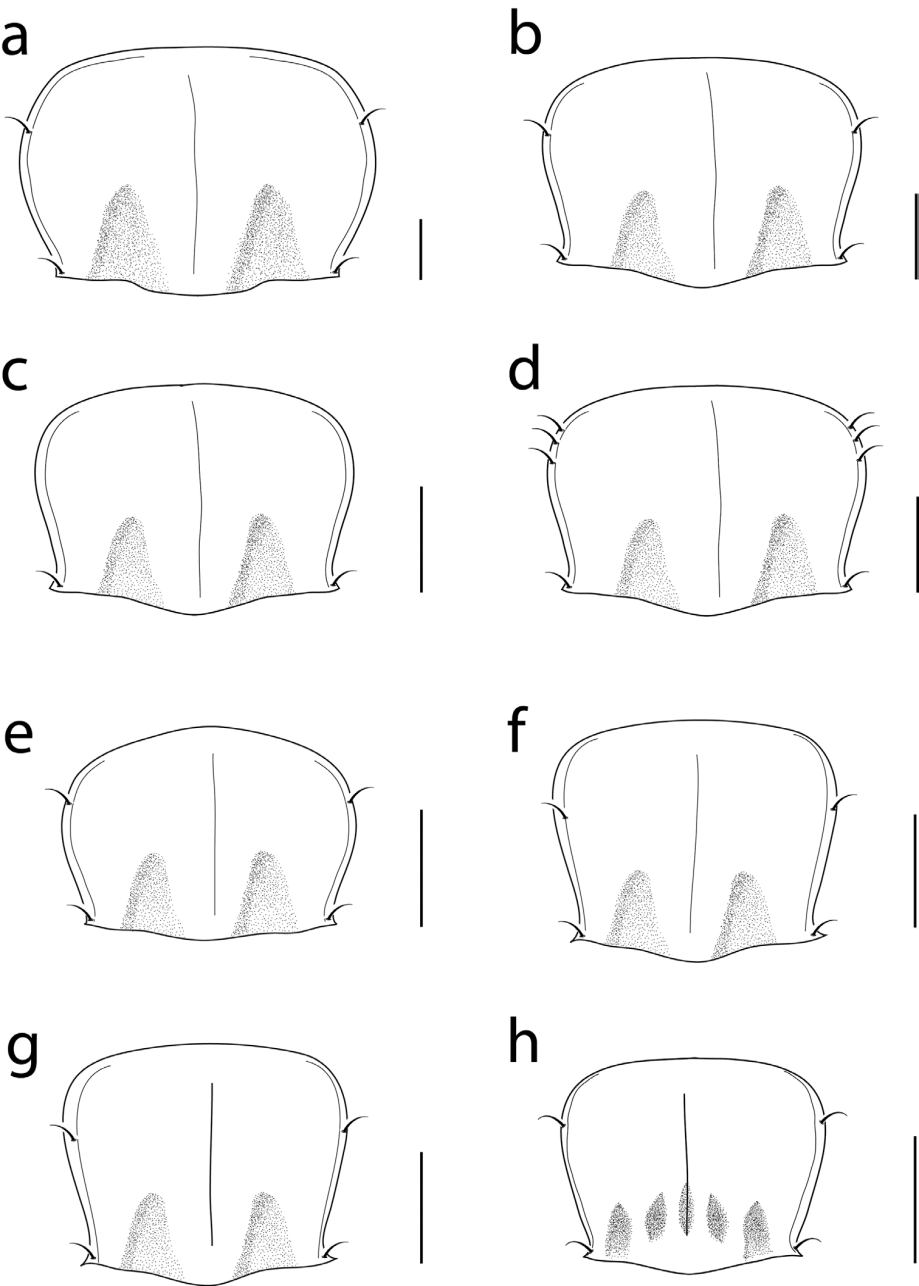
Pronotal drawings for: **a** *Cratocerus monilicornis* **b** *Cratocerus sulcatus* **c** *Cratocerus sinesetosus* **d** *Cratocerus multisetosus* **e** *Cratocerus tanyae* **f** *Cratocerus indupalmensis* **g** *Cratocerus kavanaughi* and **h** *Cratocerus culpepperi*. Scale bars = 0.5 mm.

**Figure 10.**
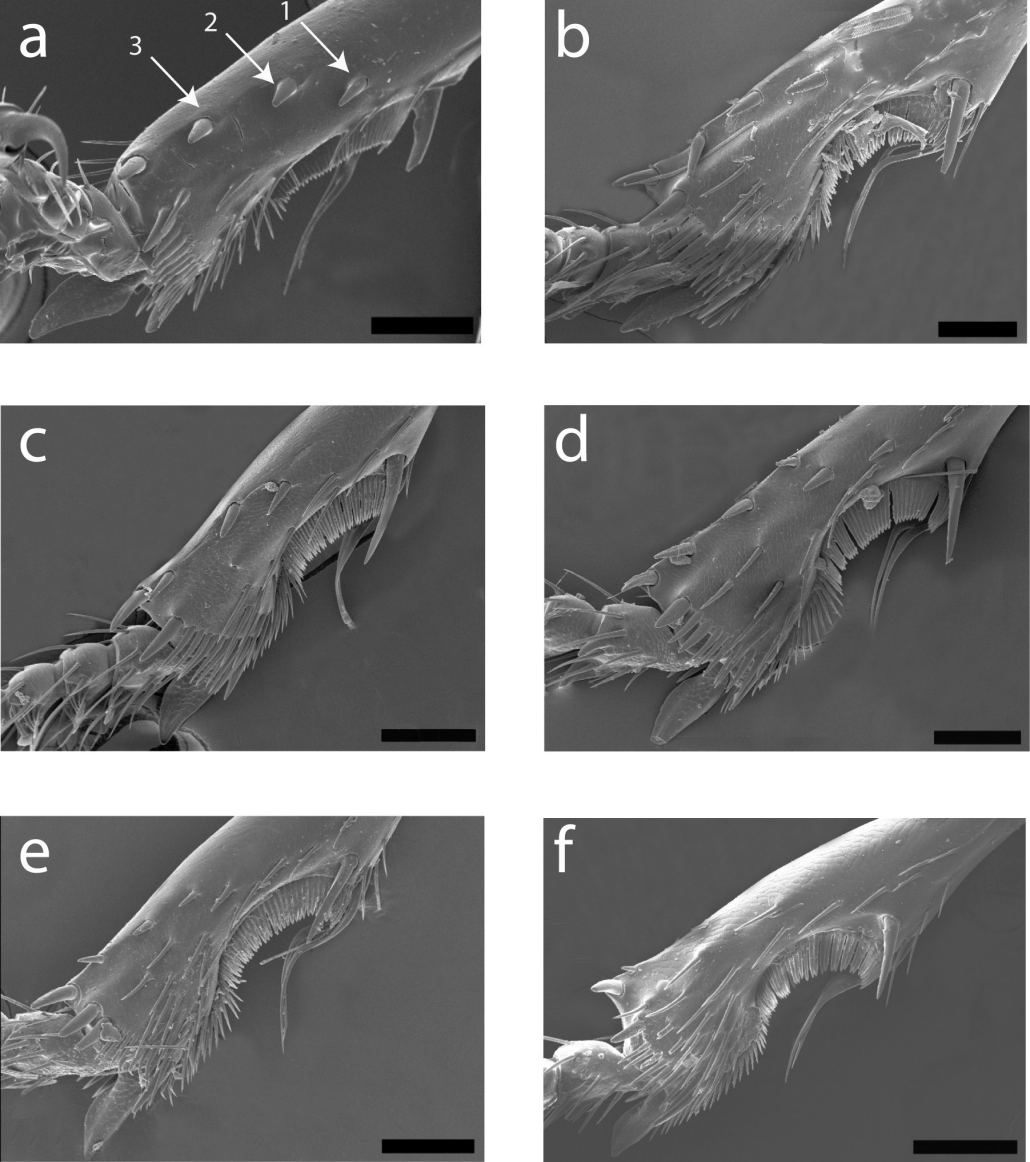
Scanning electron micrographs of left protibia for: **a** *Cratocerus sinesetosus* **b** *Cratocerus multisetosus* **c** *Cratocerus tanyae* **d** *Cratocerus indupalmensis* **e** *Cratocerus kavanaughi* **f** *Cratocerus culpepperi*. Arrows point to distinct, stout spines referred to in text. Scale bars = 100 µm.

**Figure 11.**
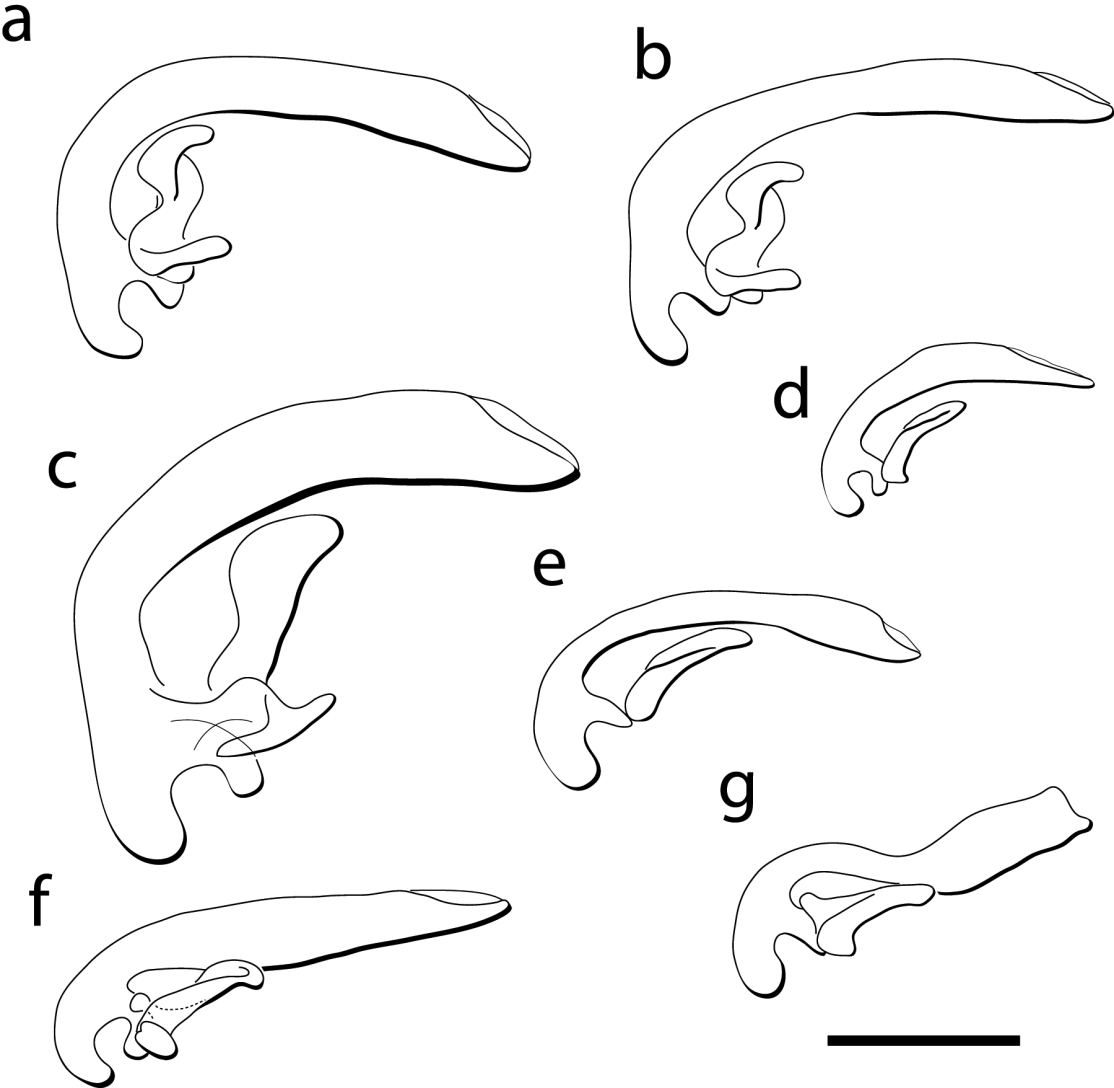
Male genitalia, left lateral view of the median lobe and left paramere for: **a** *Cratocerus sinesetosus* **b** *Cratocerus multisetosus* **c** *Cratocerus monilicornis*; **d** *Cratocerus tanyae* **e** *Cratocerus indupalmensis* **f** *Cratocerus kavanaughi* **g** *Cratocerus culpepperi*. Scale bar = 0.5 mm.

**Figure 12.**
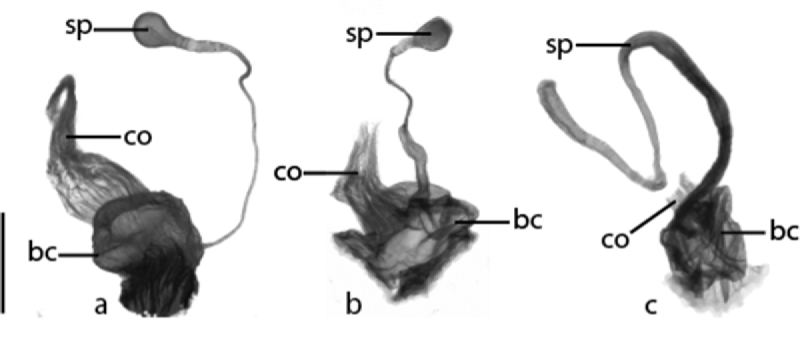
Ventral view of anterior portion of female reproductive tract. **a** *Cratocerus sinesetosus* **b** *Cratocerus culpepperi* and **c** *Cratocerus kavanaughi*. Abbreviations: bc, bursa copulatrix; co, common oviduct; sp, spermatheca. Scale bar = 0.25 mm.

Figs 8 , 9h , 10f , 11g , 12b

#### Type locality.

Peru, Loreto region, Rio Napo, Explomapo Camp, Rio Sucusari, coordinates 3.25S, 72.92W.

#### Type material.

HOLOTYPE [male, deposited in UNMSM] labeled: “PERU: LORETO Rio Napo / Explomapo Camp, Rio Sucusari/ 100m, 6 June 1992 / 03°15'S, 072°55'W, / T.L. Erwin, E. & F. Pfuno S. // Insecticidal fog of suspended / dry leaves in low canopy and one / tree to 35m(14 sheets) along / Tr. Pestoso in Igapo 500m from / camp Lot 337 A // ADP / 53163 // U.C. Berkeley / EMEC 207,967”.

**Paratypes.** 8 females with same data as the holotype (EMEC207960, EMEC207961, EMEC207962, EMEC207964, EMEC207965, EMEC207966, EMEC207968, EMEC207969), deposited in NMNH. 2 males, labeled: PERU: “LORETO Rio Napo Explomapo Camp, Rio Sucusari 100m, 15 June 1992 03°15'S, 072°55'W, T.L. Erwin, E. & F. Pfuno S. Insecticidal fog of 20m high canopy mixed with dry leaves (8m3)(14 sheets), with 1 sheet nearly pure Guadua (dry and green leaves) Lot 387” (EMEC207963, EMEC654858). All paratype specimens deposited in NMNH except EMEC207960 deposited in UNMSM.

#### Diagnosis.

This species is distinguished from congeners by the small overall body size, light brunneous coloration, the five basal pronotal fovea, and the mandibles curved more than 45 degrees in relation to the mandibular midline. This species most closely resembles *Cratocerus kavanaughi*, but is easily distinguished by the smaller size and the five basal pronotal fovea as opposed to two.

#### Description.

Size small for the genus, ABL 3.5–4.0 mm, TW 1.4–1.6 mm. *Color*. Head, labrum, mandibles, prothorax, elytra, and abdominal sternites castaneous; remaining mouthparts, antennae, and legs testaceous. *Head*. HL 0.4–0.6 mm, HW 0.5–0.7 mm. One supraorbital seta present over each eye. Apices of mandibles strongly curved, apices at more than 45 degree angle to mandibular midline. Apical maxillary palpomere length 3.5 × width at base. Mentum with triangular lateral lobes. *Thorax*. Pronotum (Fig. 9h) with distinct median impression, two distinct basal fovea laterad and one basal fovea centered on median impression; one lateral seta located on apicolateral pronotal bead; one posterior seta present on posterior bead and projecting over posterior angle; pronotal anterior margin convex, greatest width anterad of center; pronotal width 1.1–1.2 × pronotal length. Elytral length 1.5–1.6 × combined elytral width. *Legs*. Protibia with one small, stout spine confined to distal half of lateral margin, only visible with extreme magnification (Fig. 10f). *Genitalia, male*. Median lobe curved sharply, between forty-five and ninety degrees with respect to basal 1/3, remaining 2/3 with continuing curvature; expanded apically; length average for genus. Left paramere curved and simple (Figure 11g). *Genitalia, female*. Spermatheca with 1/3 length moderately thin, following 1/3 length extremely thin and filamentous, gradually expanding approximately 1/3 below apex, ended with broad bulb (Fig. 12b).

#### Etymology.

This species is named in recognition of Meghan Culpepper, currently a PhD student of carabid phylogenetics and biogeography who provided endless amounts of support throughout the duration of this project. Though this species is one of the smallest within the genus *Cratocerus*, the red hue of the integument and morphological characters mentioned above make it one of the most distinctive. Miss Culpepper is equally unique and we name this species for her.

#### Distribution.

**Figure 13.**
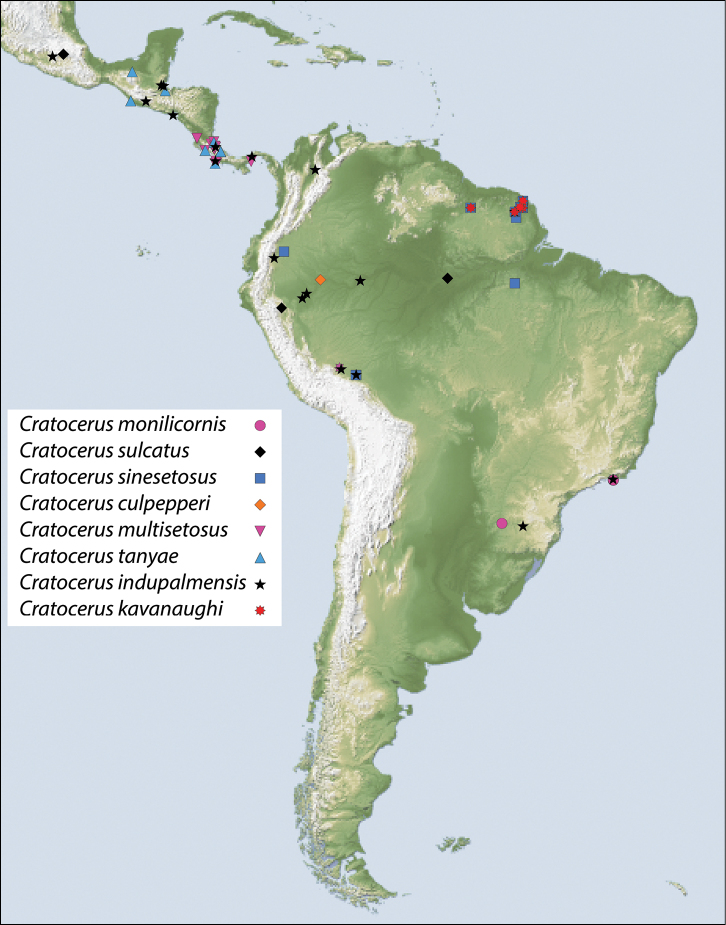
Map showing location of material examined for species of *Cratocerus*.

This species is currently only known from Peru (Fig. 13).

#### Ecology.

Specimens examined in this study were all collected from insecticidal fogging of suspended dry leaves in the canopy and a tree during the month of June.

### Key to the species of *Cratocerus* Dejean

**Table:** 

1	Two pairs of supraorbital setae; pronotum widest at midpoint; larger species, ABL 9.5–12.0 mm; habitus as in Figure 1; Venezuela, Brazil–northeast Argentina	*Cratocerus monilicornis*
–	One pair of supraorbital setae; pronotum widest anterad of middle; smaller species, ABL 3.5–6.5 mm	2
2	Pronotum with two, occasionally three, pairs of anterior marginal setae (Fig. 9d); habitus as in Figure 4; Costa Rica–Panama	*Cratocerus multisetosus* sp. n.
–	Pronotum with one or no pairs of anterior marginal setae (Fig. 9b, c, e–h)	3
3	Apices of mandibles strongly curved, forming an approximately right angle with the mandibular midline	4
–	Apices of mandibles slightly curved, forming less than 45 degree angle to the mandibular midline	5
4	Outer and inner pair of pronotal basal fovea present (Fig. 9h); small in size, ABL 3.5–4.0 mm; habitus as in Figure 8; Peru	*Cratocerus culpepperi* sp. n.
–	Outer pair of pronotal basal fovea present, inner pair absent (Fig. 9g); midsized, ABL 4.8–5.6 mm; habitus as in Figure 7; French Guiana and Peru	*Cratocerus kavanaughi* sp. n.
5	Pronotum without anterior marginal setae (Fig. 9c); habitus as in Figure 3; French Guiana, Peru, and Ecuador	*Cratocerus sinesetosus* sp. n.
–	Pronotum with one pair of anterior marginal setae (Fig. 9b, e, f)	6
6	Pronotum wider than long (Fig. 9b); larger specimens, ABL 5.7–6.1 mm, maximum elytral width 2.7 mm; habitus as in Figure 2; males with aedeagus as in Fig. 11b; Mexico, Brazil, Peru	*Cratocerus sulcatus*
–	Pronotum approximately as wide as long (Fig. 9e and f); smaller specimens, ABL 4.1–5.5 mm, maximum elytral width 2.4 mm	7
7	Smaller in size, ABL 4.1–4.7 mm; pronotum widest just anterad of center (Fig. 9e); males with aedeagus as in Fig. 11d; habitus as in Figure 5; Mexico–Costa Rica	*Cratocerus tanyae* sp. n.
–	Larger size, ABL 4.9–5.5 mm; pronotum widest near anterior apices (Fig. 9f); males with aedeagus as in Fig. 11e; habitus as in Figure 6; Mexico–Brazil	*Cratocerus indupalmensis* sp. n.

## Discussion

No detailed study of the characteristics of *Cratocerus* relative to their possible placement in a tribal scheme has been done. They are clearly placed in Harpalinae based on the shared harpalidian abdominal configuration (Liebherr and Will 1998, Deuve 1993). An association with *Catapiesis*, *Brachidius* and *Oxyglychus* was suggested by Will et al. (2000) given similarities of tergite VIII, whereby the tergite is divided into hemitergites and those further divided into epitergites in a manner unlike lebiomorphs and orthogonines. The tergal configuration in *Cratocerus*, *Catapiesis*, *Brachidius* and *Oxyglychus* is not equivalent to “tergite turrets” of lebiomorphs and orthogonines (contra Erwin 1984, Erwin 1985, Bousquet 2012, Ober and Maddison 2008).

Decyl acetate is an uncommonly produced compound in carabids that was found in the defensive chemicals of the pygidial glands in *Catapiesis* species (Will et al. 2000) and has also been found in *Brachidius* (Will and Attygalle unpublished data). Pygidial gland compounds are not known for *Cratocerus* and *Oxyglychus*. Preliminary analyses of three independent molecular loci consistently group *Cratocerus*, *Catapiesis*, and *Brachidius* (Will, Kanda and Maddison unpublished data), but no sequence data are available for *Oxyglychus*. Though preliminary and incomplete, these new findings are consistent with recognition of Cratocerini sensu Lorenz (2005), and the placement of the tribe in Harpalinae remains *sedis mutabilis*.

## Supplementary Material

XML Treatment for Cratocerus

XML Treatment for Cratocerus monilicornis

XML Treatment for Cratocerus sulcatus

XML Treatment for Cratocerus sinesetosus

XML Treatment for Cratocerus multisetosus

XML Treatment for Cratocerus tanyae

XML Treatment for Cratocerus indupalmensis

XML Treatment for Cratocerus kavanaughi

XML Treatment for Cratocerus culpepperi
